# Efficient rhizobium strains enhance nitrogen fixation and growth in alfalfa by improving photosynthetic carbon metabolism and respiratory nitrogen assimilation

**DOI:** 10.1186/s12870-026-08954-4

**Published:** 2026-05-26

**Authors:** Wenjuan Kang, Yuanyuan Du, Wenlu Hou, Yilin Han, Baofu Lu, Jian Guan, Shangli Shi

**Affiliations:** Key Laboratory of Grassland Ecosystem (Gansu Agricultural University), Ministry of Education, Pratacultural College, Gansu Agricultural University, Lanzhou, 730070 China

**Keywords:** *Medicago sativa*, Symbiotic effects-different rhizobium, Photosynthetic metabolism, Respiratory metabolism, Energy strategy

## Abstract

**Background:**

Improved symbiotic nitrogen fixation efficiency between alfalfa (*Medicago sativa* L.) and rhizobia represents a green development strategy that addresses the demand for high‑quality protein, while also serving as a critical measure for safeguarding China’s food security. Currently, there is limited research on how rhizobium inoculation influences alfalfa growth and development through photosynthesis and respiratory metabolism. Furthermore, studies examining the impact of rhizobium strains with differing symbiotic effectiveness on these metabolic pathways remain scarce.

**Results:**

The number of effective nodules per plant (7), nitrogenase activity (0.29 µmol·g^− 1^·h^− 1^), and leghemoglobin content (0.76 mg·g^− 1^) of the LL2 inoculation group were significantly higher than those of the QL5 group. The aboveground dry weight (0.59 g·10 plants^− 1^) of LL2 was also significantly greater than that of both the QL5 inoculation treatment and the uninoculated control. These results demonstrate that rhizobium strain LL2 is an efficient symbiotic match for ' Gannong No.9 ' alfalfa, whereas strain QL5 is an inefficient match. Metabolomic analysis revealed that, in leaves, seven differential metabolites were up-regulated in both photosynthetic and respiratory metabolism. Among these, Adenosine 5’-Diphosphate (ADP) was significantly higher in LL2 than in CK (Control) and QL5. In roots, nine differential metabolites were up-regulated. Among these, four metabolites—3-Phosphoglyceric acid, Uridine-5’-diphosphate-glucose, (2 S)-2-Isopropylmalate, and L-Glutamic acid—were present at significantly higher levels in LL2 than in both CK and QL5. Compared to the QL5 group, the LL2 inoculation group resulted in significantly higher contents of ADP in leaves and elevated levels of the root metabolites such as the photosynthetic carbon fixation intermediate 3-Phosphoglyceric acid, the glycosyl donor Uridine-5’-diphosphate-glucose, the respiration and nitrogen metabolism-related compounds (2S)-2-Isopropylmalate and L-Glutamic acid. Additionally, in nodules, the key metabolites trehalose-6-phosphate and alpha-D-glucose-6-phosphate (involved in sugar metabolism and the pentose phosphate pathway) were also significantly elevated Among these, ADP and alpha-D-glucose-6-phosphate participate simultaneously in photosynthetic, respiratory, and symbiotic metabolic pathways; 3-Phosphoglyceric acid is involved in both photosynthetic and symbiotic pathways; while (2S)-2-Isopropylmalate and L-Glutamic acid take part in respiratory and symbiotic pathways.

**Conclusions:**

Following inoculation with LL2, the levels of key metabolites associated with photosynthesis and respiration underwent systematic changes in the leaves, roots, and nodules of the plants. The enhanced symbiotic nitrogen fixation and plant growth were associated with synergistic changes in the host plant’s photosynthetic carbon metabolism, respiratory energy metabolism, and nitrogen assimilation pathways. The findings of this study suggest potential strategies for enhancing nitrogen accumulation, possibly through modulating the energy balance of the symbiotic system, which could improve nitrogen fixation efficiency and ultimately increase legume yield and quality.

**Supplementary Information:**

The online version contains supplementary material available at 10.1186/s12870-026-08954-4.

## Introduction

Alfalfa (*Medicago sativa* L.) is a perennial leguminous forage crop widely cultivated in China [1] and serves as an essential high‑quality feed source for the livestock industry [2]. Rhizobia can infect the roots of legumes such as alfalfa, to form nodules, and fix atmospheric nitrogen—which plants cannot directly utilize—into forms that provide nitrogen nutrition to the host plant [3]. In return, the plant supplies energy for nodule development and nitrogen fixation. The symbiotic nitrogen fixation process is highly energy‑intensive, requiring approximately 16 molecules of ATP to reduce one molecule of N_2_ [4]. By promoting alfalfa growth, increasing yield, and improving forage quality [5], rhizobial inoculation can reduce dependence on chemical nitrogen fertilizers, enhance soil fertility, and contribute to energy conservation and environmental protection [6]. Fully realizing the nitrogen‑fixing potential of rhizobia is therefore of significant importance for sustainable agricultural development.

Photosynthesis and respiration exhibit a close synergistic interaction, forming a bidirectional metabolic cycle of raw materials and products. Through photosynthesis, green plants convert CO_2_ and H_2_O into high-energy organic compounds while releasing O_2_ [7]. Conversely, respiration oxidizes organic matter back into CO_2_ and H_2_O, releasing energy to sustain life processes. Thus, photosynthesis and respiration are both antagonistic and interdependent, together constituting the core metabolic network that underpins plant life activities [8].

Photosynthesis, one of the most crucial chemical reactions on Earth, not only supplies energy for plants themselves but also releases oxygen, providing essential oxygen resources for other organisms [9]. Through the carbon-oxygen balance, it helps maintain the stability of Earth’s ecosystems. As the basis of plant autotrophy, photosynthesis enables plants to convert inorganic substances into organic matter to meet their own growth and developmental needs [10]. Respiration, on the other hand, is a vital life process in which organic compounds within cells are gradually oxidized and decomposed under enzymatic catalysis, thereby releasing energy [11]. This energy supports various plant life activities such as growth, movement, and cellular metabolism, making respiration indispensable for sustaining life and development [12].

The study found that under natural light conditions, the biomass, chlorophyll content and photosynthetic capacity of soybean were significantly increased after inoculation with rhizobia [13]. Compared with the uninoculated treatment, the inoculated soybean plants were higher, and their nitrogen content, chlorophyll content, net photosynthetic rate and seed yield were significantly increased [14].

Ma et al. [15] found that rhizobial inoculation significantly increased chlorophyll content, net photosynthetic rate (*Pn*), and stomatal conductance (*Gs*) in leaves of two soybean varieties. Nodule number, nodule weight per plant, aboveground and belowground biomass were also significantly enhanced, along with notable increases in sucrose content, leghemoglobin content, and nitrogenase activity within nodules. Wu et al. [16] demonstrated that single inoculation with *Glomus etunicatum* or *Sinorhizobium fredii* led to increases in chlorophyll content, Pn, succinate dehydrogenase activity, mitochondrial membrane H⁺-ATPase activity, biomass, and yield in soybean. The dual inoculation treatment exerted a synergistic effect that was significantly superior to either single inoculation, resulting in improved nodulation rate and nitrogen fixation efficiency. This enhanced nutrient absorption in host plants, promoted the growth of intercropped soybeans, and ultimately led to greater biomass and yield. It was also reported that in ‘Xinmu No. 1’ alfalfa, inoculation with alfalfa rhizobia or soybean rhizobia resulted in higher Pn, Gs, transpiration rate (*Tr*), and water use efficiency compared to the uninoculated control (CK) [17]. Similarly, Liu et al. [18] observed that under natural light conditions, inoculation with strain SD101 significantly increased aboveground biomass, leaf chlorophyll content, and photosynthetic efficiency in plants.

Numerous studies have demonstrated that rhizobial inoculation enhances plant photosynthetic capacity, as evidenced by increased *Pn*, *Gs*, intercellular CO_2_ concentration (Ci), and chlorophyll content. Respiration-derived energy serves as the fundamental basis for plant metabolism, providing the driving force for various physiological activities and playing a critical role in plant growth and development [19]. Within plants, energy is primarily stored in the form of adenosine triphosphate (ATP). As the central energy carrier in living organisms, ATP represents the most direct form of energy supply for life activities. ATP synthase plays a key role in the terminal reaction of oxidative phosphorylation in mitochondria [20, 21]. Wu et al. found that after inoculation with Sinorhizobium, ATP synthase activity in soybean leaves increased, and its variation pattern was consistent with that of the net photosynthetic rate. However, little research has focused on how respiratory metabolism changes in alfalfa under symbiotic nitrogen fixation conditions.

However, physiological data alone are insufficient to deeply analyze the effects of rhizobial inoculation on plant photosynthesis and respiration. As a pivotal component of systems biology, metabolomics enables the qualitative and quantitative analysis of all metabolites within an organism. Metabolites represent the endpoint of biochemical reactions and serve as the ultimate expression of gene and protein activity, directly reflecting the phenotypic state of an organism. Through metabolomic studies, we can gain deep insights into metabolic changes under various physiological and environmental conditions, thereby providing key information for uncovering the intrinsic mechanisms of biological processes.

In summary, current studies on photosynthesis and respiration in rhizobium-inoculated legumes have predominantly focused on physiological parameters such as *Pn*, *Gs*, *Ci*, and *Tr*, while research on corresponding photosynthetic and respiratory metabolites remains limited. Moreover, although numerous studies on alfalfa have examined growth and developmental changes under symbiotic nitrogen fixation, little attention has been given to its photosynthetic and respiratory metabolism under these conditions. Furthermore, the differential impact of rhizobium strains with varying symbiotic efficiencies on photosynthesis and respiration has not been systematically investigated. Therefore, it is necessary to analyze how different rhizobium inoculations influence plant photosynthesis, respiratory metabolism, nodulation capacity, and overall growth. Such research will be crucial for enhancing photosynthetic efficiency in symbiotic alfalfa, optimizing the energy strategy of the alfalfa–rhizobium symbiotic system, and ultimately improving nitrogen accumulation, yield, and quality in alfalfa production.

## Materials and methods

### Experimental materials

*Sinorhizobium meliloti* LL2 and *Sinorhizobium meliloti* QL5 were isolated from the root nodules of *M. sativa* ‘Longzhong’ and *M. sativa* ‘Qingshui’, respectively. They were sequenced and identified by the Microbial Identification and Preservation Center of the Chinese Academy of Sciences and are currently preserved at the Key Laboratory of Grassland Ecosystem (Ministry of Education), Gansu Agricultural University. *M. sativa* ' Gannong No.9 ' was provided by the Key Laboratory of Pratacultural Ecosystem, Ministry of Education, Gansu Agricultural University.

The reagents used in the experiment include methanol, acetonitrile, formic acid, etc., all of which are chromatographically pure. The specific information is shown in Table 1. The experimental instruments include mass spectrometer, ultra-high performance liquid chromatography and other equipment. Details are shown in Table 2.

**Table 1:** Experimental reagents

Name	CAS numbering	Purity	Brand
Methanol	67-56-1	Chromatographically Pure	Merck
Acetonitrile	75-05-8	Chromatographically Pure	Shanghai Xingke
Formic acid	64-18-6	Chromatographically Pure	Aladdin

**Table 2:** Experimental equipment

Name	Model	Brand	Habitat
Mass spectrometer	TripleTOF 6600+	SCIEX	Foster City, CA, USA
Liquid Chromatograph	LC-30 A	Shimadzu	Japan
Centrifuge	5424R	Eppendorf	Hamburg, Germany
metal mixer	MU-G02-0448	Hangzhou Miou Instrument Co., Ltd	Hangzhou, China
Electronic balances	MS105DM	Metter-Toledo Instruments Co., Ltd	Zurich, Switzerland
Centrifugal concentrator	CentriVap	LABCONCO	Missouri Kansas, USA
vortex mixer	VORTEX-5	Kyllin-Bell	Haimen, China
Ultrasonic cleaning instrument	KQ5200E	Kunshan Ultrasonic Instruments Co., Ltd	Kunshan, China
Pipettor	Research plus	Eppendorf	Hamburg, Germany
Automation workstation	Biomek i5	Beckman Coulter	Califomia, USA

### Seed sterilization and seedling cultivation

The experiment was conducted using a nutrient solution sand culture method in an artificial climate chamber. The growth chamber was set to the following environmental parameters: a light intensity of 360 µmol·m^−^²·s^−^¹, relative humidity maintained at 60% ± 5%, a day/night temperature regime of 25 °C (light period) and 20 °C (dark period), and a photoperiod of 16 h light / 8 h dark.

Full and uniformly sized *M. sativa '* Gannong No.9 ' seeds were selected for the study. The seeds were surface-sterilized by immersing in a 0.45%–0.55% (w/v) iodophor solution for 3 minutes, followed by repeated rinsing with sterile water. Subsequently, the seeds were soaked in ST solution (a mixture of 0.9% sterile sodium chloride and 0.5% Tween 80) for 1 minute, washed again with sterile water, and dried with filter paper [22]. Fine sand (The particle size is between 0.5 and 2.0 mm) was washed, sterilized by dry-heat at 120°C for 0.5 hours, and allowed to cool.

This study established three replicated treatments to evaluate rhizobial inoculation effects on alfalfa (‘ Gannong No.9 ‘). Six pots per replicate were assigned to: (1) inoculation with strain LL2, (2) inoculation with strain QL5, and (3) sterile water control (CK). After 45 days of growth, nodulation and nitrogen fixation parameters were assessed.

The sterilized seeds were evenly sown in mesh culture cups (9 cm in diameter, 12 cm in height) containing a 1:3 mixture of sterilized sand and perlite (horticultural grade perlite, with a particle size of 3 to 6 mm). Cups were placed in hydroponic boxes measuring 25 cm × 15 cm × 10 cm (L × W × H), with six cups per box and 20 seeds per cup. All materials were then transferred to the growth chamber for cultivation.

### Strain activation and bacterial suspension preparation

The cryopreserved strains LL2 and QL5 were first streaked onto TY solid medium [23] (containing tryptone: 5 g·L^−^¹, yeast extract: 3 g·L^−^¹, CaCl_2_·6H_2_O: 1.3 g·L^−^¹, agar: 16 g·L^−^¹; prepared in 1000 mL distilled water) and incubated until single colonies appeared. Selected colonies were then transferred into YMA liquid medium (composed of MgSO_4_·7H_2_O: 0.2 g·L^−^¹, K_2_HPO_4_·3H_2_O: 0.5 g·L^−^¹, NaCl: 0.1 g·L^−^¹, mannitol: 10 g·L^−^¹, yeast extract: 1 g·L^−^¹; pH 7.0; prepared in 1000 mL distilled water) and cultured at 28 °C with shaking at 180 r·min^−^¹ for 18 h.

After cultivation, the bacterial cells were harvested by centrifugation at 10,000 r·min^−^¹ for 10 min at 4 °C. The supernatant was discarded, and the pellet was washed with an equal volume of sterile water and resuspended thoroughly to obtain a uniform bacterial suspension with an OD_600nm_ of 0.5 (corresponding to approximately 10^9^ CFU·mL^−^¹) [24].

### Inoculation treatment

Fourteen days after seedling emergence, 5 mL of bacterial suspension was pipetted directly onto the roots of each seedling [25]. An equal volume of sterile water was applied separately as a control (CK). Each treatment was replicated with three hydroponic boxes.

After inoculation, 300 mL of nitrogen‑free Hoagland nutrient solution was added to each pot and replaced every three days. When alfalfa plants reached approximately 45 days of age, samples were collected for determination of nodulation, nitrogen‑fixation capacity, phenotypic traits, photosynthesis, respiration, and related physiological indices.

### Sample collection and processing

On the 45th day of growth, symbiotic and control (CK) plants were cleaned to remove surface impurities. Using a sterile scalpel, leaves, nodules, and roots from symbiotic plants, as well as leaves and roots from CK plants, were rapidly excised and transferred into 10 mL cryotubes. The samples were immediately frozen in liquid nitrogen for 15 min and then stored at − 80 °C for subsequent analysis. Each sample type was prepared with three biological replicates, each weighing over 0.3 g, to provide sufficient material for non‑targeted metabolomics. Sample details and abbreviations are listed in Table 3.

**Table 3:** Sample Information

Treatments	Organs	Abbreviations
Inoculation of rhizobium LL2	Leaf	LL2L
	Root	LL2R
	Root nodule	LL2RN
Inoculation of rhizobium QL5	Leaf	QL5L
	Root	QL5R
	Root nodule	QL5RN
Inoculation of the same amount of sterile water	Leaf	CKL
	Root	CKR

### Measurement of nodulation, nitrogen fixation, and phenotypic traits

#### Number of effective nodules per plant

Ten alfalfa plants were randomly selected from each treatment. After rinsing off surface impurities, the number of effective nodules per plant was counted under a microscope (effective nodules were identified by their light‑pink coloration). The procedure was performed in three replicates per treatment.

#### Single nodule fresh weight

Ten alfalfa plants were randomly chosen, and their roots were gently washed. Nodules were carefully detached, surface‑dried with filter paper, and immediately weighed on an electronic balance to determine fresh weight. Three replicates were carried out per treatment.

#### Nodule diameter

Thirty nodules of similar developmental stage were randomly selected and measured for diameter.

#### Nitrogenase activity

Nitrogenase activity was assessed using the acetylene reduction assay [26]. Fresh nodules were quickly excised, weighed, and transferred into 8 mL penicillin bottles. The bottles were sealed, and 0.8 mL of air was replaced with an equal volume of acetylene gas using a microsyringe. After incubation at room temperature for 2 h, 50 µL of headspace gas was sampled with a 100 µL microsyringe and injected into a gas chromatograph (Agilent 7890B) for analysis. Each treatment was analyzed in three replicates.

#### Leghemoglobin content

Leghemoglobin content was determined according to a previously described method [23]. Absorbance was measured at 540 nm using a TU‑1901 spectrophotometer, with three replicates per treatment.

#### Nitrogen fixation potential per plant

This parameter was calculated as:

$$\text{Nitrogenase activity}\times\text{nodule fresh weight per plant}.$$

Five plants per treatment replicate were randomly harvested. Aboveground biomass and underground biomass: The plant samples were separated into aboveground and underground parts. The aboveground parts were killed out at 105 °C for 0.5 h and then dried at 70 °C to a constant weight. The underground parts were carefully washed to remove soil, and then dried using the same procedure. The dry weight of each part was recorded.

### Non-targeted metabolomics analysis

(1) Dry sample extraction: Biological samples were lyophilized in a Scientz‑100 F freeze‑dryer [27] for 63 h under vacuum. The dried samples were ground into a fine powder using a mill (Retsch). Subsequently, 50 mg of the powder was weighed, and 1200 µL of a pre‑cooled (–20 °C) 70% methanol‑water internal standard extraction solution was added. The internal standard solution was prepared by dissolving 1 mg of the standard compound in 1 mL of 70% methanol‑water to obtain a 1000 µg·mL^−^¹ stock solution, which was then diluted with 70% methanol to a working concentration of 250 µg·mL^−^¹. The mixture was vortexed for 30 s every 30 min, for a total of six cycles. After vortexing, the sample was centrifuged, and the supernatant was passed through a microporous membrane filter prior to UPLC‑MS/MS analysis.

(2) T3 chromatographic conditions: A Waters ACQUITY UPLC HSS T3 column (1.8 μm particle size, 2.1 mm × 100 mm) was used. Mobile phase A consisted of ultrapure water containing 0.1% formic acid, and mobile phase B was acetonitrile containing 0.1% formic acid. The column temperature was maintained at 40 °C, the flow rate was set to 0.40 mL/min, and the injection volume was 4 µL. Tables 4 and 5 show the specific T3 column mobile phase gradient conditions and AB TripleTOF 6600 mass spectrometry conditions, respectively.

**Table 4:** T3 column mobile phase gradient conditions

Time (min)	A (%)	B (%)
0.0	95	5
5.0	35	65
6.0	1	99
7.5	1	99
7.6	95	5
10.0	95	5

**Table 5:** AB TripleTOF 6600 mass spectrometry conditions

name	ESI+	ESI-
Duration (min)	10	10
IonSpray Voltage (V)	5000	-4000
Temperature (°C)	550	550
Ion Source Gas1 (psi)	50	50
Ion Source Gas2 (psi)	60	60
Curtain Gas (psi)	35	35
Declustering Potential (V)	60	-60
MS1 Collision Energy (V)	10	-10
MS2 Collision Energy (V)	30	-30
Collision Energy Spread (V)	15	15

### Statistic analysis

Data were organized and calculated using Microsoft Office 2019 software, with graphs generated via Prism. Statistical analyses were performed using SPSS software. One-way analysis of variance (ANOVA) was used for comparisons among multiple groups, followed by Tukey’s post hoc test for pairwise comparisons. Processing of Metabolomics Data: Raw mass spectrometry data were first converted to mzML format using ProteoWizard tools. Subsequently, XCMS software was employed for peak extraction, peak alignment, and retention time correction. Chromatographic peaks with a missing rate exceeding 50% across all sample groups were excluded. A hybrid imputation strategy was applied to missing values: when the missing rate was > 50%, values were imputed with 1/5 of the minimum value; when the missing rate was ≤ 50%, K-nearest neighbor (KNN) imputation was used. Metabolites with a composite score ≥ 0.5 and a coefficient of variation (CV) < 0.5 in quality control (QC) samples were ultimately selected, and data from positive and negative ion modes were merged. Differential metabolites were screened based on three criteria: (1) VIP > 1, indicating a strong contribution to group discrimination; (2) fold change ≥ 2 or ≤ 0.5; and (3) *p* < 0.05, denoting statistical significance.

## Results

### Differential effects of rhizobium strains on alfalfa symbiosis and growth

**Fig. 1:**
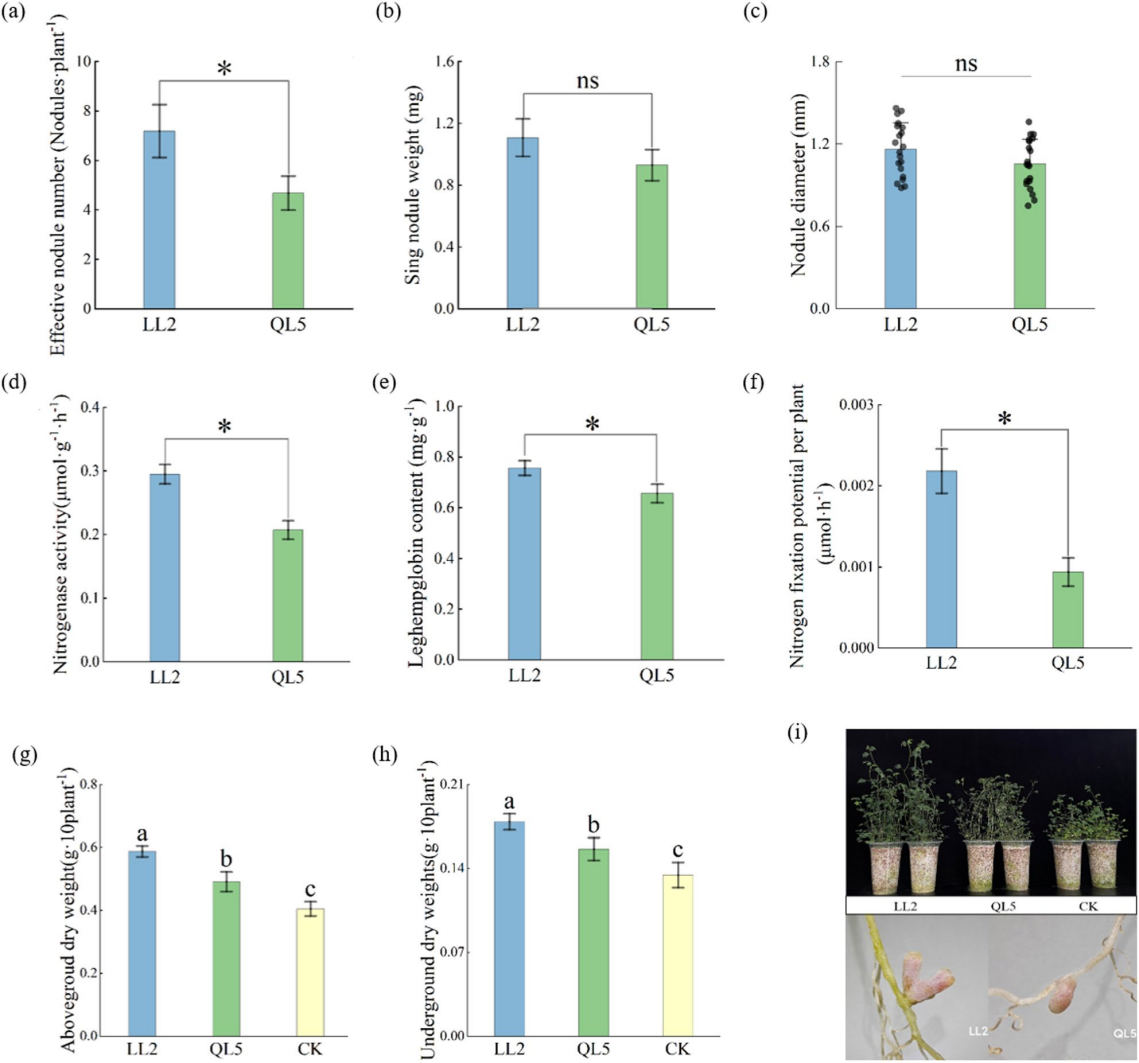
Effects of rhizobial strains with different symbiotic efficiencies on the nodulation, nitrogen fixation capacity, and plant biomass of alfalfa. * *p* <0.05; ns means not significant, Same below. Among them, (**a**) is the number of effective nodules per plant, (**b**) is the weight of a single nodule, (**c**) is the diameter of nodules, (**d**) is nitrogenase activity, (**e**) is leghemoglobin content, (**f** )is nitrogen fixation potential per plant, (**g**) is the ground dry weight, (**h**) is the underground dry weight and (**i**) is the experimental diagram of inoculated culture and two types of root nodule diagrams

As shown in Fig. 1, the effective nodule number per plant of ‘Gannong No. 9’ alfalfa inoculated with rhizobium LL2 (7) was significantly higher than that with strain QL5 (4) (*p* < 0.05) (Fig. 1a). The LL2 treatment resulted in 1.75 times more effective nodules than QL5. While there were no significant differences in single nodule weight (Fig. 1b) or nodule diameter (Fig. 1c) between the two treatments, both parameters showed a consistent trend of being higher in the LL2 group.

Gannong No. 9 alfalfa inoculated with rhizobium LL2 had significantly higher nitrogenase activity than the QL5 treatment (0.29 vs. 0.21 µmol·g^−^¹·h^−^¹, *p* < 0.05). This value was 1.38 times that of the QL5 group (Fig. 1d). The leghemoglobin content was also significantly greater in the LL2 group (0.76 vs. 0.66 mg·g^−^¹, *p* < 0.05), representing a 0.15-fold increase over QL5 (Fig. 1e). Furthermore, the nitrogen fixation potential per plant was 0.002 µmol·h^−^¹ in the LL2 treatment, compared to 0.001 µmol·h^−^¹ in the QL5 treatment (*p* < 0.05). The LL2 value was 1.34 times higher than that of QL5 (Fig. 1f).

As shown in Fig. 1g and h, rhizobium inoculation significantly increased the biomass of ‘Gannong No. 9’ alfalfa (*p* < 0.05). The LL2 treatment produced an aboveground dry weight of 0.59 g·10 plants^− 1^, which was 47.50% higher than the CK (0.40 g·10 plants^− 1^), For the QL5 treatment, the aboveground dry weight reached 0.49 g·10 plants^− 1^, a 22.50% increase over CK. (Fig. 1g). The LL2 underground dry weight was 0.18 g·10 plants^− 1^, representing a 33.09% increase over the CK (0.13 g·10 plants^− 1^), while the underground dry weight of QL5 was 0.16 g·10 plants^− 1^, which was 16.07% higher than that of CK (Fig. 1h). Between the two inoculation treatments, LL2 showed significantly higher biomass than QL5. The aboveground dry weight of LL2 was 1.20 times that of QL5, and its underground dry weight was 1.15 times that of QL5.

Fig. 1i shows the planting experiment images inoculated with two different bacteria and the comparison images of root nodules formed by the two rhizobia. 

### Organ-specific differential metabolites and KEGG enrichment analysis

#### Differential metabolites in leaves

**Fig. 2:**
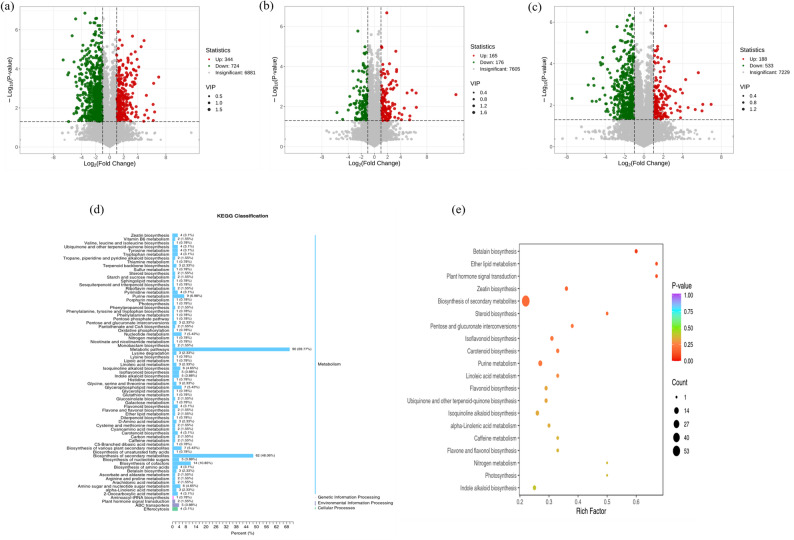
Volcano plots (**a**, **b**, **c**), pathway classification (**d**) and enrichment analysis (**e**) of differential metabolites in leaves. In volcano plots (**a**) LL2L vs. CKL, (**b**) QL5L vs. CKL and (**c**) LL2L vs. QL5L, each point corresponds to a metabolite. Green points indicate down‑regulated differential metabolites, red points indicate up‑regulated differential metabolites, and gray points represent metabolites that were detected but did not show a significant difference. The x‑axis represents log₂(fold change), i.e., the logarithm of the relative content ratio of a metabolite between two sample groups. A larger absolute x‑axis value indicates a greater difference in relative content between the two groups. Under the VIP + FC + *P*‑value screening criterion: The y‑axis represents the significance level of the difference (–log₁₀P‑value). The size of the point corresponds to the VIP value. Under the VIP + FC screening criterion: The y‑axis directly represents the VIP value. A higher y‑axis value indicates a more significant difference and greater reliability of the identified differential metabolite. The same conventions apply to subsequent plots. Pathway Classification and Enrichment of Leaf Differential Metabolites. The (**d**) panel illustrates the classification of differential metabolic pathways in LL2L vs. CKL leaves. The y‑axis indicates the names of the metabolic pathways, while the x‑axis shows both the number of differential metabolites annotated to each pathway and their percentage relative to the total annotated differential metabolites. The (**e**) panel presents the enrichment analysis of differential metabolic pathways in LL2L vs. CKL leaves. In this plot, the x‑axis represents the Rich Factor for each pathway, and the y‑axis lists the pathway names sorted by *P*‑value. The color of each point reflects the P‑value magnitude, and the point size corresponds to the number of enriched differential metabolites. The same conventions apply to the following figures

As shown in Fig. 2a, LL2L vs. CKL showed 1686 differential metabolites. These metabolites were divided into 21 categories. Amino acids and their derivatives accounted for 30.62%, organic acids for 11.62%, benzene and derivatives for 11.39%, and alkaloids for 4.63%. Other types together represented 12.76%. Among all differential metabolites, 344 were upregulated and 724 were downregulated.

A total of 928 differential metabolites were identified in QL5L vs. CKL, categorized into 20 types. Among these, amino acids and their derivatives represented 30.64%, organic acids 11.33%, benzene and its derivatives 9.49%, and glycerophospholipids 5.93%. Other metabolite types together accounted for 12.62%. In terms of regulation, 165 metabolites were upregulated and 176 were downregulated (Fig. 2b).

As shown in Fig. 2c, a total of 1327 differential metabolites were identified in LL2L vs. QL5L. These metabolites were classified into 20 categories. Among them, amino acids and their derivatives accounted for 30.54%, benzene and its derivatives for 11.61%, organic acids for 11.31%, and flavonoids for 4.45%. Other classes together represented 13.20%. In terms of regulation, 183 metabolites were upregulated and 533 were downregulated.

Differential metabolites in LL2L vs. CKL were primarily annotated to pathways associated with photosynthesis and respiratory metabolism. These included starch and sucrose metabolism, carotenoid biosynthesis, photosynthesis, pentose phosphate pathway, and oxidative phosphorylation pathway (Fig. 2d). KEGG enrichment analysis further showed that differential metabolites in LL2L vs. CKL were mainly involved in betaine biosynthesis, ether lipid metabolism, plant hormone signal transduction, zeatin biosynthesis, and biosynthesis of secondary metabolites. Additionally, pathways directly related to photosynthesis and respiration—such as carotenoid biosynthesis and photosynthesis—were also significantly enriched (Fig. 2e).

The differential metabolites in QL5L vs. CKL were mainly annotated to starch and sucrose metabolism and the pentose phosphate pathway (Supplementary Fig. 1a). KEGG enrichment analysis showed that these differential metabolites were primarily involved in glycerophospholipid metabolism, diterpenoid biosynthesis, endocytosis, autophagy-other, and vitamin B6 metabolism (Supplementary Fig. 1b).

The differential metabolites of LL2L vs. QL5L were mainly annotated in fructose and mannose metabolism, carotenoid biosynthesis, oxidative phosphorylation, pentose phosphate pathway, and photosynthesis pathway (Supplementary Fig. 1c). KEGG enrichment analysis of these metabolites showed significant involvement in arachidonic acid metabolism, α-linolenic acid metabolism, betaine biosynthesis, linoleic acid metabolism, and isoflavone biosynthesis. Additionally, pathways directly related to photosynthesis and respiratory metabolism—carotenoid biosynthesis and photosynthesis—were also notably enriched (Supplementary Fig. 1d).

#### Differential metabolites in root

**Fig. 3:**
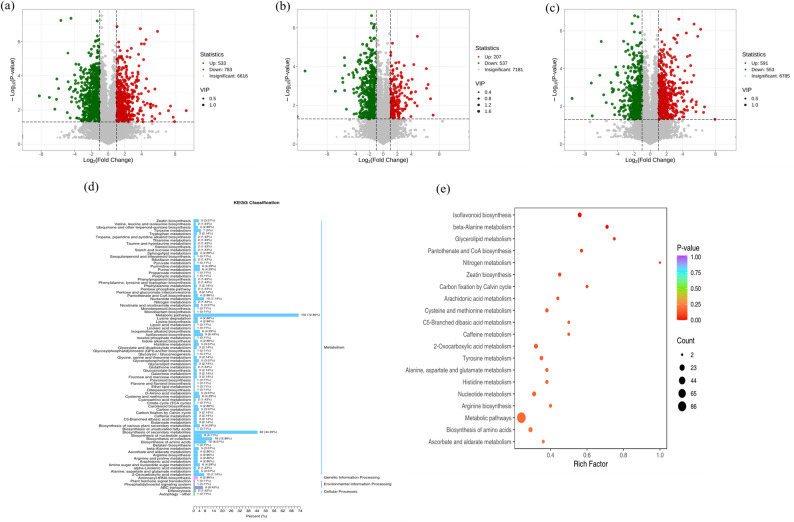
Volcano plots of roots differential metabolites in LL2R vs. CKR (**a**), QL5R vs. CKR (**b**), LL2R vs. QL5R (**c**), and pathway classification (**d**) and enrichment analysis (**e**) in LL2R vs. CKR

A total of 1907 differential metabolites were identified in LL2R vs. CKR, classified into 21 categories (Fig. 3a). Among these, amino acids and their derivatives accounted for 30.01%, organic acids for 12.49%, benzene and its derivatives for 11.69%, and alkaloids for 4.14%. Other types together represented 11.23%. According to Fig. 3a and 533 metabolites were upregulated and 783 were downregulated.

A total of 1373 differential metabolites were identified in QL5R vs. CKR, divided into 21 categories (Fig. 3b). Among these, amino acids and their derivatives accounted for 36.85%, benzene and its derivatives for 9.61%, organic acids for 9.03%, and alkaloids for 5.39%. Other types together represented 11.22%. As shown in Fig. 3b and 207 metabolites were upregulated and 537 were downregulated.

A total of 1741 differential metabolites were identified in LL2R vs. QL5R, classified into 19 categories (Fig. 3c). Among these, amino acids and their derivatives accounted for 29.70%, organic acids for 13.04%, benzene and its derivatives for 10.74%, and alkaloids for 4.54%. Other categories together accounted for 11.49%. Under the screening criteria shown in Fig. 3c and 591 metabolites were upregulated and 553 were downregulated.

The differential metabolites in LL2R vs. CKR were primarily annotated to pathways associated with photosynthesis and respiratory metabolism (Fig. 3d). These included fructose and mannose metabolism, carotenoid biosynthesis, carbon fixation in the Calvin cycle, starch and sucrose metabolism, glycolysis, the tricarboxylic acid cycle, the pentose phosphate pathway, and pyruvate metabolism. KEGG enrichment analysis further showed that differential metabolites were mainly involved in isoflavone biosynthesis, β-alanine metabolism, glycerolipid metabolism, and pantothenate and CoA biosynthesis (Fig. 3e). Notably, carbon fixation in the Calvin cycle—a pathway directly related to photosynthesis and respiration—was also significantly enriched.

The differential metabolites in QL5R vs. CKR were mainly annotated to pathways related to photosynthesis and respiration. These include fructose and mannose metabolism, carotenoid biosynthesis, glycolysis, and pyruvate metabolism pathways (Supplementary Fig. 2a). KEGG enrichment analysis showed that the differential metabolites in QL5R vs. CKR primarily participated in carotenoid biosynthesis, ether lipid metabolism, steroid biosynthesis, arachidonic acid metabolism, and glycerophospholipid metabolism (Supplementary Fig. 2b). In addition to carotenoid biosynthesis, other pathways directly related to photosynthesis and respiration were also significantly enriched. These include fructose and mannose metabolism, as well as pyruvate metabolism.

The differential metabolites of LL2R vs. QL5R were mainly annotated in pathways related to photosynthesis and respiration. These include starch and sucrose metabolism, carbon fixation in the Calvin cycle, carotenoid biosynthesis, glycolysis, pentose phosphate pathway, and pyruvate metabolism pathway (Supplementary Fig. 2c). The KEGG enrichment of these metabolites was mainly observed in carotenoid biosynthesis, nucleotide metabolism, isoflavone biosynthesis, caffeine metabolism, and glycerolipid metabolism (Supplementary Fig. 2d). The carotenoid biosynthesis pathway is directly involved in photosynthetic metabolism.

#### Differential metabolites in root nodule

**Fig. 4:**
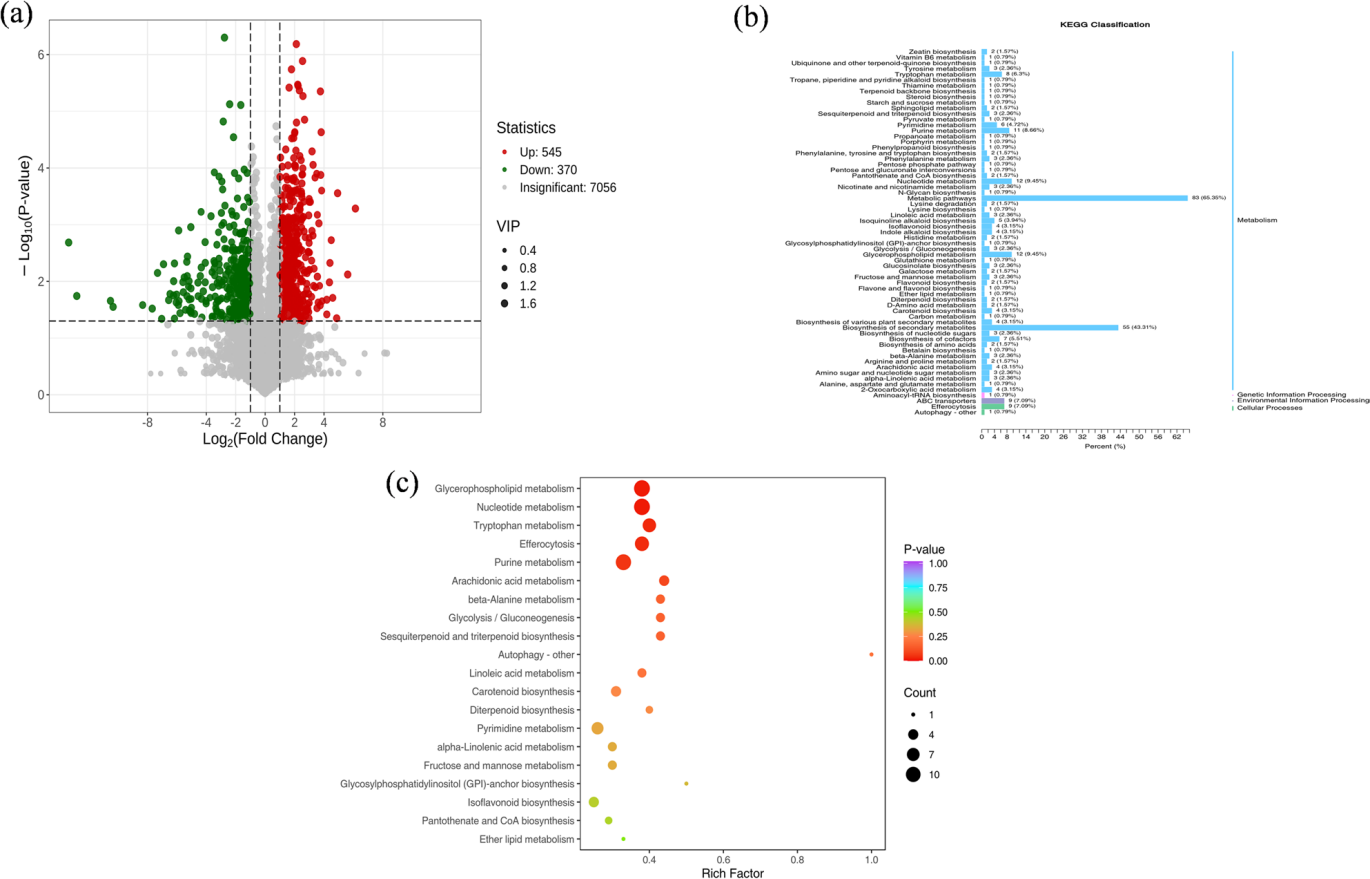
Visualizations of nodule differential metabolites between two inoculated groups (LL2RN vs. QL5RN). **a** Volcano plot. **b** Pathway classification. **c** Enrichment analysis

As shown in Fig. 4a, a total of 1621 differential metabolites were identified in LL2RN vs. QL5RN, classified into 21 categories. Among these, amino acids and their derivatives accounted for 29.92%, organic acids for 12.34%, benzene and its derivatives for 10.98%, and glycerophospholipids for 4.69%. Other categories together accounted for 11.10%, 545 metabolites were upregulated and 370 were downregulated.

As shown in Fig. 4b, differential metabolites in LL2RN vs. QL5RN were mainly annotated to pathways related to photosynthesis and respiration. These included fructose and mannose metabolism, starch and sucrose metabolism, carotenoid biosynthesis, glycolysis, pentose phosphate pathway, and pyruvate metabolism. KEGG enrichment analysis (Fig. 4c) revealed that metabolites in LL2RN vs. QL5RN were primarily enriched in nucleotide metabolism, glycerophospholipid metabolism, tryptophan metabolism, endocytosis, and purine metabolism. Additionally, pathways directly linked to photosynthesis and respiration, such as glycolysis, carotenoid biosynthesis, and fructose and mannose metabolism, were also significantly enriched.

### Comparative metabolomic analysis of symbiotic nitrogen fixation in alfalfa leaves, roots, and nodules under different rhizobial inoculation

**Fig. 5:**
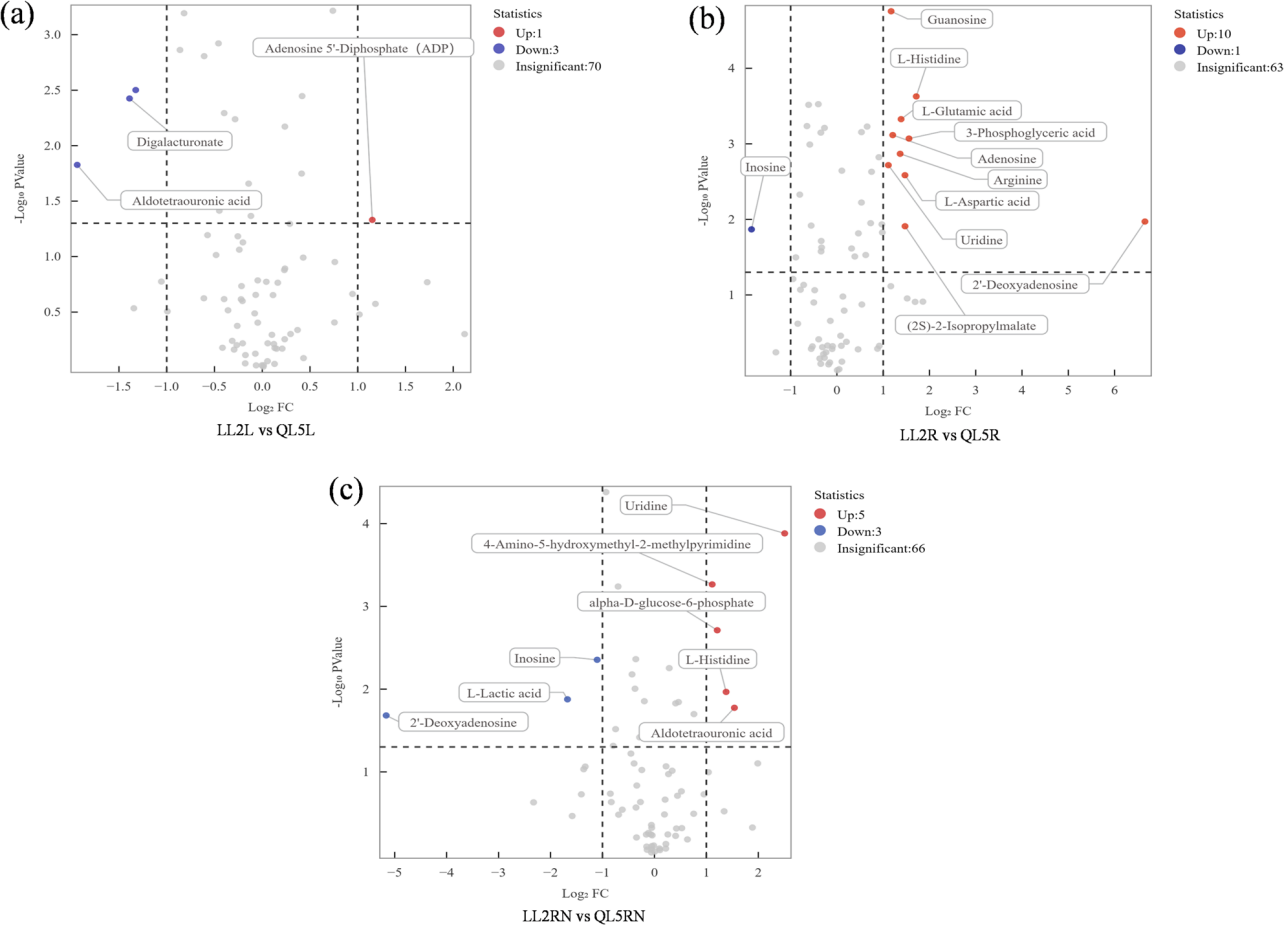
Symbiotic nitrogen fixation-related metabolites in leaves (**a**, LL2L VS QL5L), roots (**b**, LL2R VS QL5R), and nodules (**c**, LL2RN VS QL5RN). Each point in volcano plots represents a metabolite, in which the blue point represents the down-regulated differential metabolite, the red point represents the up-regulated differential metabolite, and the gray point represents the detected but not significantly different metabolite; the abscissa represents the logarithm of the relative content difference multiple of a metabolite in the two groups of samples (log_2_FC). The larger the absolute value of the abscissa, the greater the relative content difference of the metabolite between the two groups of samples. Under the FC + *P*-value screening condition, the ordinate represents the significant difference level (-log_10_*P*-value). The same below

In LL2L vs. QL5L, ADP—a key metabolite associated with nitrogen fixation—was significantly higher in LL2-treated leaves compared to QL5-treated leaves (*p* < 0.05) (Fig. 5a).

Analysis of root metabolites revealed that multiple symbiotic nitrogen fixation related compounds were significantly more abundant in LL2‑treated roots than in QL5‑treated roots (*p* < 0.05). These included 3‑Phosphoglyceric acid, L‑aspartic acid, L‑Glutamic acid, (2 S)‑2‑Isopropylmalate, L‑Histidine, Arginine, 2’‑Deoxyadenosine, Adenosine, Guanosine, and Uridine (Fig. 5b).

Similarly, in nodules (LL2RN vs. QL5RN), the concentrations of several symbiotic nitrogen fixation related metabolites were significantly higher in the LL2 treatment group. These metaboli-tes were alpha‑D‑Glucose‑6‑Phosphate, L‑Histidine, 4‑Amino‑5‑hydroxymethyl‑2‑methylpyrimidine, Aldotetraouronic acid, and Uridine (*p* < 0.05) (Fig. 5c).

### Differential metabolite analysis in photorespiratory pathways among leaves, roots, and nodules of rhizobia with contrasting symbiotic efficiency

#### Effects of rhizobial strains on photosynthetic and respiratory metabolism in leaves

**Fig. 6:**
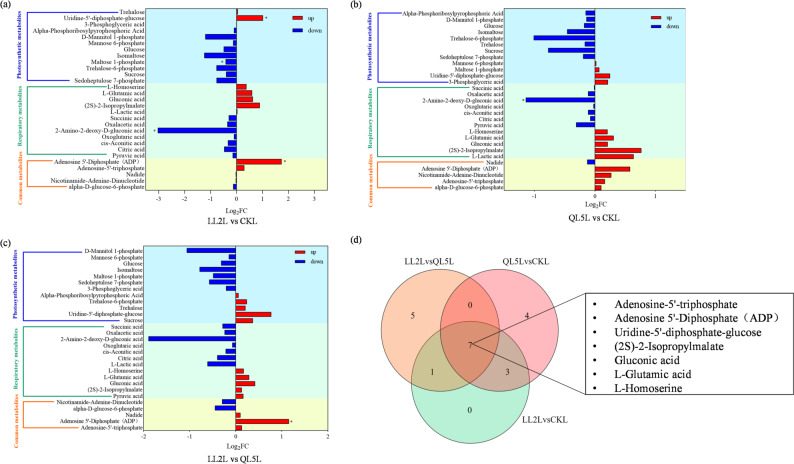
Differential metabolic pathways of photosynthesis and respiration in leaves. **a** LL2L VS CKL. **b** QL5L VS CKL. **c** LL2L VS QL5L. **d** Venn diagram of differential metabolic pathways of each group. In the bar chart, the ordinate is the name of the metabolite, the abscissa is the Log_2_FC value, and * represent significant at *p* < 0.05. The same below

In the photosynthetic and respiratory metabolic pathways of LL2L vs. CKL, several metabolites were upregulated (Fig. 6a). These include Adenosine‑5′‑triphosphate, ADP, 3‑Phosphoglyceric acid, Uridine‑5′‑diphosphate‑glucose, Trehalose, L‑Lactic acid, (2 S)‑2‑Isopropylmalate, Gluconic acid, L‑Glutamic acid, and L‑Homoserine. Among them, ADP (a common metabolite in both photosynthesis and respiration) and Uridine‑5′‑diphosphate‑glucose (a photosynthetic metabolite) showed significant upregulation (*p* < 0.05).

Analysis of Fig. 6b shows that in the photosynthetic and respiratory metabolic pathways of QL5L vs. CKL, the upregulated metabolites included alpha‑D‑glucose‑6‑phosphate, Adenosine‑5′‑triphosphate, Nicotinamide‑Adenine‑Dinucleotide, ADP, 3‑Phosphoglyceric acid, Uridine‑5′‑diphosphate‑glucose, Maltose‑1‑phosphate, Mannose‑6‑phosphate, L‑Lactic acid, (2 S)‑2‑Isopropylmalate, Gluconic acid, L‑Glutamic acid, and L‑Homoserine.

In the photosynthetic and respiratory metabolic pathways of LL2L vs. QL5L, the upregulated metabolites included Adenosine‑5′‑triphosphate, ADP, NAD⁺, Sucrose, Uridine‑5′‑diphosphate‑glucose, Trehalose, Trehalose‑6‑phosphate, α‑Phosphoribosylpyrophosphate, Pyruvic acid, (2 S)‑2‑Isopropylmalate, Gluconic acid, L‑Glutamic acid, and L‑Homoserine. Among these, the photosynthetic metabolite ADP was significantly upregulated (*p* < 0.05) (Fig. 6c).

Ten common up‑regulated differential metabolites of photosynthetic and respiratory metabolism were identified in both LL2L vs. CKL and QL5L vs. CKL leaves. Eight differential metabolites were consistently up‑regulated in the photosynthetic and respiratory pathways of LL2L vs. CKL and LL2L vs. QL5L leaves. Seven common up‑regulated differential metabolites associated with photosynthetic and respiratory metabolism were identified in both QL5L vs. CKL and LL2L vs. QL5L leaves. Seven differential metabolites were consistently up‑regulated across all three leaf comparisons (LL2L vs. CKL, QL5L vs. CKL, and LL2L vs. QL5L). Among these, ADP—a metabolite common to both photosynthesis and respiration—was significantly higher in LL2L compared to both CKL and QL5L (*p* < 0.05) (Fig. 6d).

#### Effects of rhizobial strains on photosynthetic and respiratory metabolism in roots

**Fig. 7:**
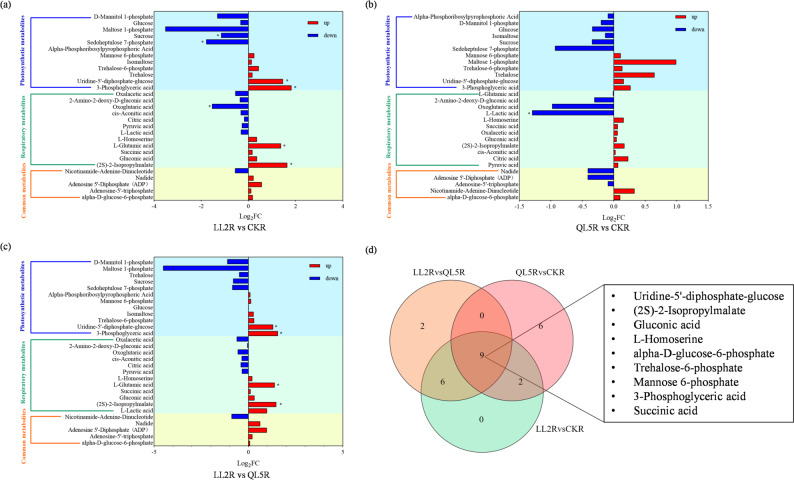
Differential metabolic pathways of photosynthesis and respiration in roots. **a** LL2R VS CKR. **b** QL5R VS CKR. **c** LL2R VS QL5R. **d** Venn diagram of differential metabolic pathways of each group

As shown in Fig. 7a, in the photosynthetic and respiratory metabolic pathways of LL2R vs. CKR, the upregulated metabolites included alpha‑D‑glucose‑6‑phosphate, Adenosine‑5′‑triphosphate, ADP, NAD⁺, 3‑Phosphoglyceric acid, Uridine‑5′‑diphosphate‑glucose, Trehalose, Trehalose‑6‑phosphate, Isomaltose, Mannose‑6‑phosphate, Alpha-Phosphoribosylpyrophosphoric Acid, (2 S)‑2‑Isopropylmalate, Gluconic acid, Succinic acid, L‑Glutamic acid, and L‑Homoserine. Among these, the significantly upregulated metabolites were the photosynthesis‑related compounds 3‑Phosphoglyceric acid and Uridine‑5′‑diphosphate‑glucose, as well as the respiration‑related compounds (2 S)‑2‑Isopropylmalate and L‑glutamic acid (*p* < 0.05).

In the photosynthetic and respiratory metabolic pathways of QL5R vs. CKR (Fig. 7b), the upregulated metabolites included alpha‑D‑glucose‑6‑phosphate, Nicotinamide‑Adenine‑Dinucleotide, 3‑Phosphoglyceric acid, Uridine‑5′‑diphosphate‑glucose, Trehalose, Trehalose‑6‑phosphate, Maltose‑1‑phosphate, Mannose‑6‑phosphate, Pyruvic acid, Citric acid, cis‑Aconitic acid, (2 S)‑2‑Isopropylmalate, Gluconic acid, Oxalacetic acid, Succinic acid, and L‑Homoserine (Fig. 7b).

Analysis of Fig. 7c shows that in the photosynthetic and respiratory metabolic pathways of LL2R vs. QL5R, the upregulated metabolites included alpha‑D‑glucose‑6‑phosphate, Adenosine‑5′‑triphosphate, ADP, Nadide, 3‑Phosphoglyceric acid, Uridine‑5′‑diphosphate‑glucose, Trehalose‑6‑phosphate, Isomaltose, Glucose, Mannose‑6‑phosphate, Alpha-Phosphoribosylpyrophosphoric Acid, L‑Lactic acid, (2 S)‑2‑Isopropylmalate, Gluconic acid, Succinic acid, L‑Glutamic acid, and L‑Homoserine. The significantly upregulated metabolites were the photosynthesis‑related compounds 3‑Phosphoglyceric acid and Uridine‑5′‑diphosphate‑glucose, as well as the sugar‑ and respiration‑related metabolites (2 S)‑2‑Isopropylmalate and L‑Glutamic acid (*p* < 0.05).

A total of 11 common differentially expressed metabolites were identified in both LL2R vs. CKR and QL5R vs. CKR roots (Fig. 7d). Fifteen common differentially expressed metabolites were found in both LL2R vs. CKR and LL2R vs. QL5R roots. Nine common differentially expressed metabolites were observed in both QL5R vs. CKR and LL2R vs. QL5R roots. Nine differential metabolites were consistently up‑regulated across all three root comparisons (LL2R vs. CKR, QL5R vs. CKR, and LL2R vs. QL5R). Among these, the photosynthesis‑related metabolites 3‑Phosphoglyceric acid and Uridine‑5′‑diphosphate‑glucose, as well as the respiration‑related metabolites (2 S)‑2‑Isopropylmalate and L‑glutamic acid, were significantly higher in LL2R compared to both CKR and QL5R (*p* < 0.05) (Fig. 7d).

#### Effects of rhizobial strains on photosynthetic and respiratory metabolism in root nodules

**Fig. 8:**
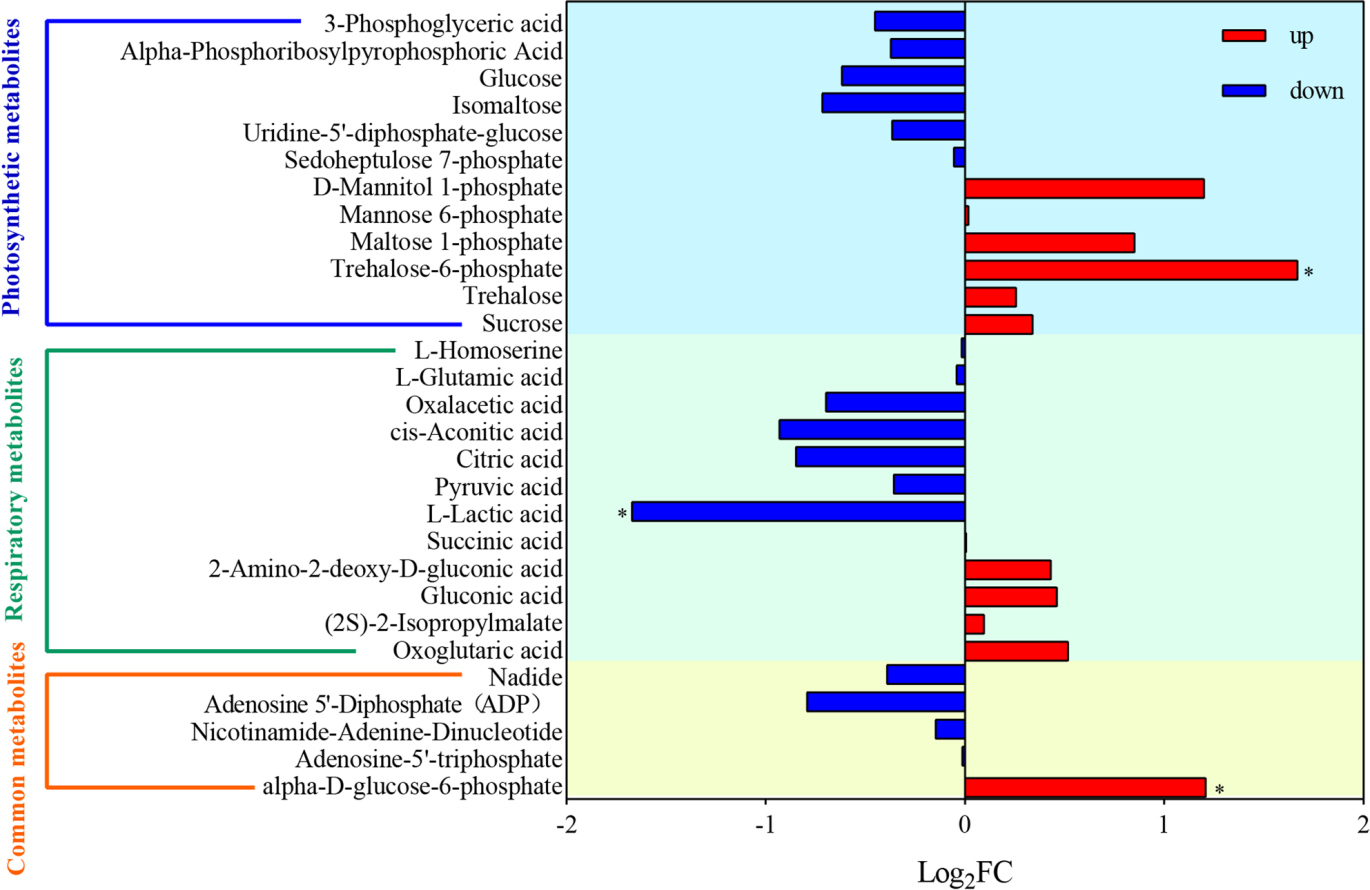
Differential metabolic pathways of photosynthesis and respiration in root nodules

As shown in Fig. 8, in the photosynthetic and respiratory metabolic pathways of LL2RN vs. QL5RN nodules, the upregulated metabolites related to photosynthesis and respiration in LL2 included alpha‑D‑glucose‑6‑phosphate, Sucrose, Trehalose, Trehalose‑6‑phosphate, Maltose‑1‑phosphate, Mannose‑6‑phosphate, D‑Mannitol‑1‑phosphate, Oxoglutaric acid, (2 S)‑2‑Isopropylmalate, Gluconic acid, 2‑Amino‑2‑deoxy‑D‑gluconic acid, and Succinic acid. Among these, the significantly upregulated metabolites were the photosynthesis‑related metabolite Trehalose‑6‑phosphate and the photosynthesis‑respiration common metabolite alpha‑D‑glucose‑6‑phosphate.

### Analysis of organ-specific differences in photosynthetic and respiratory metabolic pathways of alfalfa following rhizobial inoculation

#### Differential photosynthetic and respiratory features across organs under LL2 inoculation

**Fig. 9:**
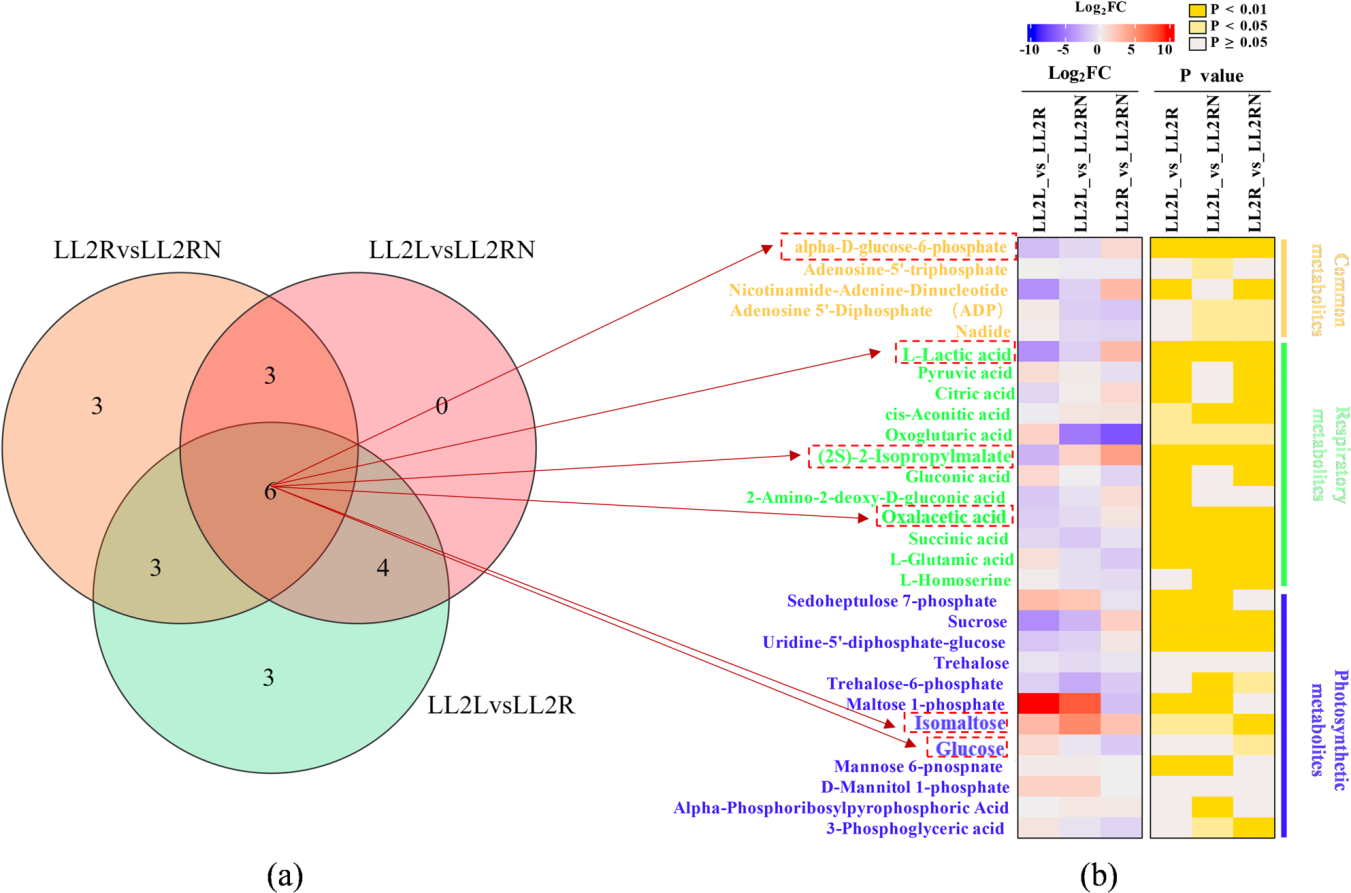
Photosynthetic and respiratory differential metabolites in various organs of LL2 inoculated plants. **a** Venn diagram of differential metabolites in each group. **b** Heatmap of differential metabolites in each group

Analysis of LL2L vs. LL2R, LL2L vs. LL2RN, and LL2R vs. LL2RN revealed six common differential metabolites in photosynthetic and respiratory metabolism across LL2 tissues (Fig. 9a). Among these, the photosynthesis-related metabolite Isomaltose was significantly more abundant in leaves than in other tissues (*p* < 0.05). The photosynthesis-related metabolite Sucrose, the respiration-related metabolites L‑Lactic acid and (2 S)‑2‑Isopropylmalate, and the common photosynthesis‑respiration metabolite alpha‑D‑glucose‑6‑phosphate were all significantly higher in roots compared to other tissues (*p* < 0.05). In nodules, the respiration-related metabolite Oxoglutaric acid was present at significantly higher levels than in leaves and roots (*p* < 0.05).

As shown in Fig. 9b and 16 metabolites were up-regulated in the photosynthetic and respiratory metabolic pathway of LL2L vs. LL2R. Significantly up-regulated metabolites included the photosynthesis-related compounds Sedoheptulose 7‑phosphate, Maltose 1‑phosphate, and Isomaltose, as well as the respiration-related compounds Pyruvic acid, Oxoglutaric acid, and Gluconic acid (*p* < 0.05). In the photosynthetic and respiratory metabolic pathway of LL2L vs. LL2RN, 11 metabolites were up-regulated. Significantly up-regulated metabolites comprised the photosynthesis-related compounds Sedoheptulose 7‑phosphate, Maltose 1‑phosphate, and Isomaltose, along with the respiration-related metabolite (2 S)‑2‑Isopropylmalate (*p* < 0.05). In the LL2R vs. LL2RN photosynthetic and respiratory metabolic pathway, 14 metabolites were up-regulated. Significantly up-regulated metabolites included the photosynthesis-related compounds Sucrose and Isomaltose; the respiration-related compounds L‑Lactic acid, Citric acid, and (2 S)‑2‑Isopropylmalate; and the photosynthesis‑respiration common metabolites alpha‑D‑glucose‑6‑phosphate and Nicotinamide‑Adenine‑Dinucleotide (*p* < 0.05).

#### Differential photosynthetic and respiratory features across organs under QL5 inoculation

**Fig. 10:**
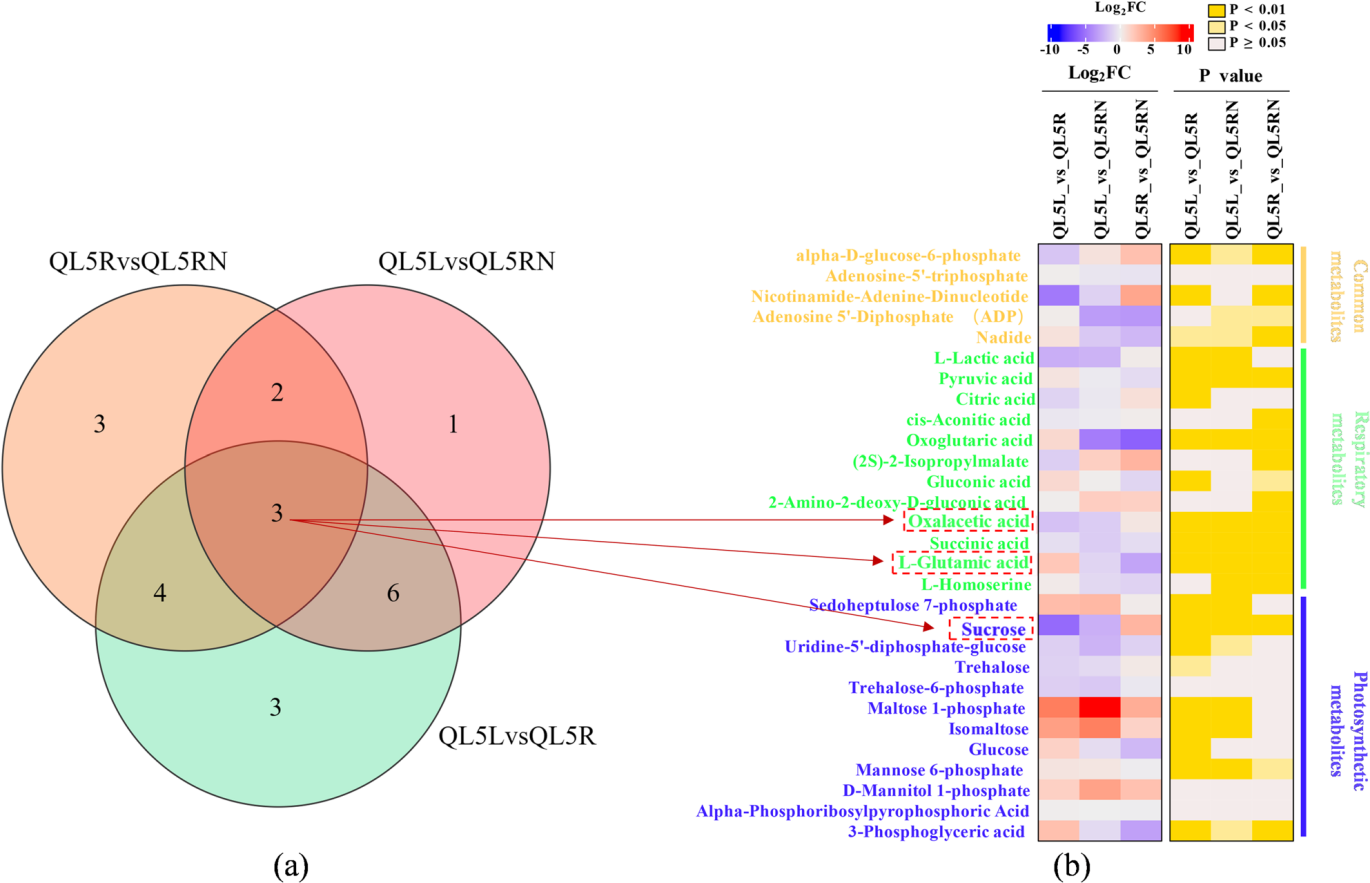
Photosynthetic and respiratory differential metabolites in various organs of QL5 inoculated plants. **a** Venn diagram of differential metabolites in each group. **b** Heatmap of differential metabolites in each group

Analysis of QL5L vs. QL5R, QL5L vs. QL5RN, and QL5R vs. QL5RN revealed three common differential metabolites in the photosynthetic and respiratory metabolism across QL5 tissues (Fig. 10a). Among these, the photosynthesis-related metabolite Sucrose was significantly more abundant in roots than in other tissues (*p* < 0.05), while the respiration-related metabolites Oxoglutaric acid and L‑Glutamic acid were significantly higher in nodules compared to other tissues (*p* < 0.05).

As shown in Fig. 10b and 17 metabolites were up‑regulated in the photosynthetic respiratory metabolism of QL5L vs. QL5R. Significantly up‑regulated metabolites included the photosynthesis‑related compounds Sedoheptulose 7‑phosphate, Maltose 1‑phosphate, Isomaltose, and 3‑Phosphoglyceric acid, as well as the respiration‑related compounds Oxoglutaric acid, Gluconic acid, and L‑glutamic acid (*p* < 0.05). In the photosynthetic respiratory metabolism of QL5L vs. QL5RN, 9 metabolites were up‑regulated. Significantly up‑regulated metabolites comprised the photosynthesis‑related compounds Sedoheptulose 7‑phosphate, Maltose 1‑phosphate, and Isomaltose (*p* < 0.05). In the photosynthetic respiratory metabolism of QL5R vs. QL5RN, 14 metabolites were up‑regulated. Significantly up‑regulated metabolites included the photosynthesis‑related compound Sucrose; the respiration‑related compounds (2 S)‑2‑Isopropylmalate and 2‑Amino‑2‑deoxy‑D‑gluconic acid; and the photosynthesis‑respiration common metabolites alpha‑D‑glucose‑6‑phosphate and Nicotinamide‑Adenine‑Dinucleotide (*p* < 0.05).

### Regulation patterns of rhizobium strains with different symbiotic effects on photosynthesis, respiration, and symbiotic nitrogen fixation in alfalfa

**Fig. 11:**
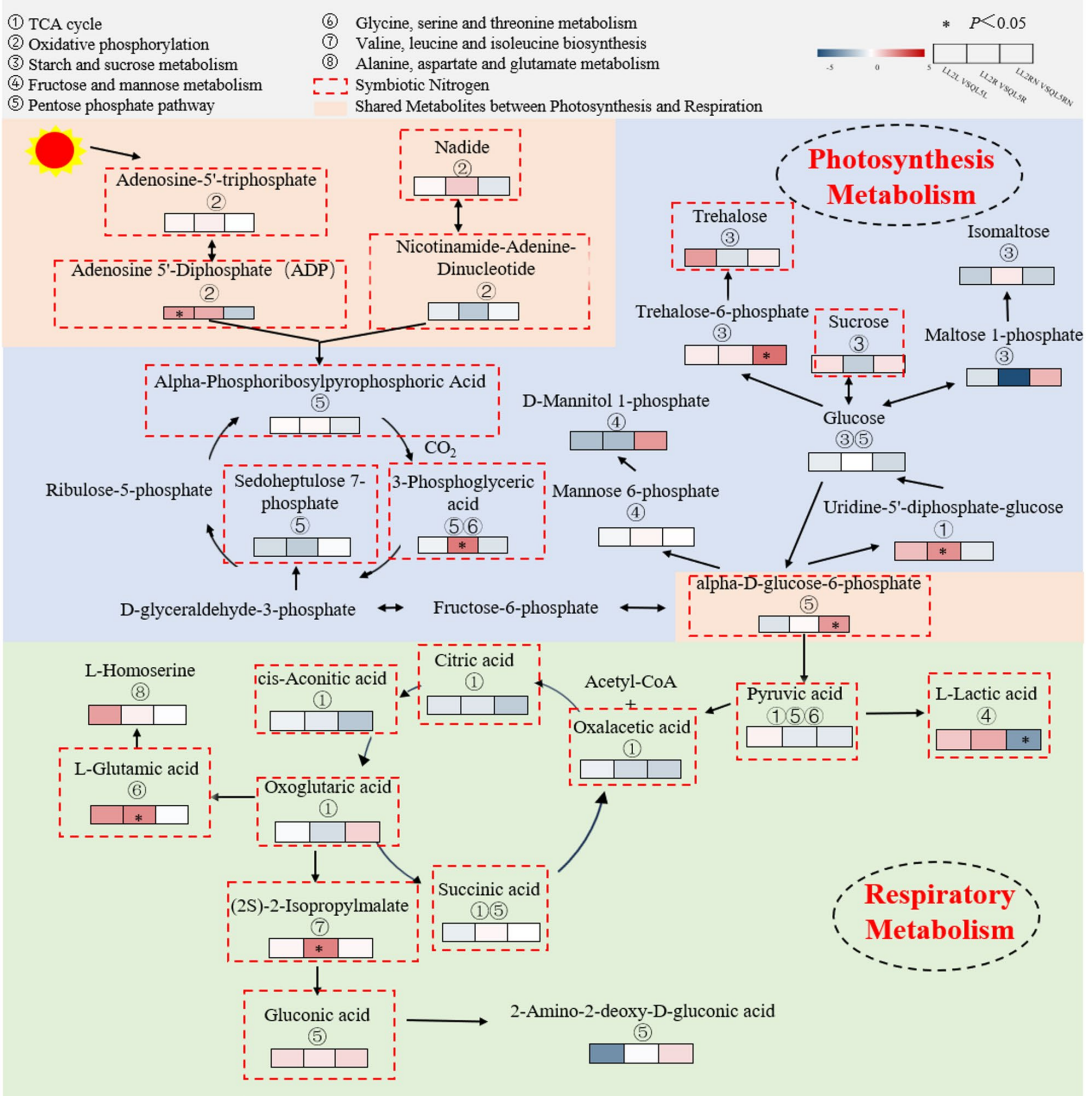
Patterns of rhizobium strains with different symbiotic effects regulating photosynthesis and respiratory metabolism of alfalfa

As indicated in Fig. 11, inoculation with the high-efficiency rhizobium strain LL2 significantly altered the levels of key metabolites involved in photosynthesis and respiration in alfalfa leaves, roots, and nodules. These metabolic changes correlate closely with the observed overall enhancement of plant growth and symbiotic nitrogen fixation capacity.

In leaves, the ADP content increased significantly by 1.15 times compared to QL5 (*p* < 0.05). This demonstrates enhanced plant energy metabolism. In roots, the content of photosynthetic intermediates also rose significantly. 3-Phosphoglyceric acid increased by 1.56 times, and Uridine-5’-diphosphate-glucose by 1.30 times (*p* < 0.05). This indicates active carbon assimilation and nucleotide sugar metabolism, which provided essential carbon skeletons and energy for nodule development and nitrogen fixation.

In nodules, the contents of Trehalose-6-phosphate and alpha-D-glucose-6-phosphate significantly increased. Compared to QL5, Trehalose-6-phosphate rose by 3.18 times, and alpha-D-glucose-6-phosphate by 2.13 times (*p* < 0.05). These two metabolites are key nodes in sugar metabolism and the pentose phosphate pathway. Alpha-D-glucose-6-phosphate is a metabolic intersection point. It links glycolysis, the pentose phosphate pathway, and glycogen synthesis. Its accumulation helps activate the pentose phosphate pathway. This activation provides reducing power (NADPH) and energy (ATP) for the nitrogenase reaction. Therefore, it directly supports the nitrogen fixation function of nodules.

The levels of respiration- and nitrogen metabolism-related metabolites, (2 S)-2-Isopropylmalate and L-Glutamic acid, were significantly increased in roots. They rose by 1.47 times and 1.39 times, respectively, compared to QL5 (*p* < 0.05). This synergistic enhancement indicates that LL2 inoculation facilitates efficient nitrogen absorption, assimilation, and transport.

The results indicate that LL2 inoculation promotes multi-pathway synergistic regulation, optimizing the overall plant metabolic network. This is evidenced by the changes in key metabolites at critical network nodes: ADP and Alpha-D-glucose-6-phosphate interconnect photosynthesis, respiration, and symbiotic metabolism; 3-Phosphoglyceric acid bridges photosynthetic and symbiotic metabolism; L-Glutamic acid and (2 S)-2-Isopropylmalate link respiratory and symbiotic metabolism.

The above metabolomics data align with the phenotypic observations. Alfalfa inoculated with strain LL2 showed significantly greater effective nodule number per plant, higher nitrogenase activity, and increased aboveground dry weight compared to plants inoculated with QL5.

In summary, the efficient rhizobium strain LL2 synergistically improves alfalfa growth and nitrogen fixation by coordinating multiple host metabolic pathways. These include photosynthetic carbon metabolism, the pentose phosphate pathway, respiratory energy metabolism, and nitrogen assimilation. This integrated metabolic response offers an important foundation for understanding the physiological basis of the rhizobia-alfalfa interaction.

## Discussion

### Differential effects of rhizobial strains on alfalfa photosynthesis and respiration

This study systematically analyzed the metabolic effects of two rhizobium strains with different symbiotic efficiencies—LL2 and QL5—on alfalfa leaves, roots, and nodules using non-targeted metabolomics. The results showed that the metabolite composition and content in various alfalfa organs changed significantly after inoculation with LL2 and QL5. Inoculation with the efficient symbiotic strain LL2 was associated with significantly altered profiles of metabolites linked to both photosynthesis and respiratory metabolism in alfalfa. This was reflected in a notable increase in the types and levels of metabolites, particularly in amino acid and organic acid metabolism.

Photosynthesis essentially converts light energy into chemical energy. Based on energy conversion, photosynthesis can be divided into three stages [28, 29]. In the primary reaction, light‑excited pigment molecules in the reaction center release high‑energy electrons, completing the conversion of light to electrical energy. These electrons are then transferred along the photosynthetic electron transport chain [30, 31]. This primary reaction leads to charge separation in the photosystem reaction center, and the resulting high‑energy electrons drive electron transfer across the photosynthetic membrane [32]. The outcomes of electron transfer include the photolysis of water—releasing oxygen and reducing NADP⁺—and the establishment of a transmembrane proton gradient, which drives photosynthetic phosphorylation to produce ATP. Thus, electrical energy is converted into active chemical energy [33]. In this study, regarding photosynthetic metabolism, the levels of photosynthesis‑related metabolites in alfalfa leaves and roots were significantly higher in the LL2 treatment group. This observed difference may be related to the higher nitrogen‑fixation capacity associated with LL2. Specifically, after LL2 inoculation, key metabolites in the starch and sucrose metabolism pathways—such as Uridine‑5′‑diphosphate‑glucose—were markedly elevated in alfalfa leaves. This suggests that the rhizobium strain enhances plant photosynthetic capacity by regulating carbohydrate metabolism, thereby increasing the allocation of photosynthetic products toward carbon storage and transport [34]. This finding aligns with previous reports that efficient rhizobial strains enhance host photosynthetic capacity by regulating carbohydrate metabolism [35]. Overall, LL2 enhances leaf photosynthetic carbon fixation and carbohydrate allocation, supplying sufficient carbon skeletons for efficient nitrogen fixation, whereas QL5 exhibits limited regulatory effects on photosynthetic metabolism. This is consistent with the findings of previous studies on the effects of rhizobial strains with different symbiotic efficiencies on the photosynthetic characteristics and respiratory metabolism of alfalfa, specifically regarding the measurement of physiological parameters such as chlorophyll concentration, photosynthetic activity, and respiration rate [36].

It should be noted that the observed differences in photosynthesis-related metabolites in this study may be influenced by variations in the number, size, or developmental stage of nodules induced by strains LL2 and QL5. However, through metabolomic analysis, we found that the accumulation of key metabolites such as Uridine-5’-diphosphate-glucose in leaves was significantly higher following LL2 inoculation compared to QL5. Based on reports by Lepetit M et al. regarding the correlation between nitrogen saturation/deficiency systemic signaling and rapid changes in nodule sugar levels, which regulate symbiosis through carbon resource allocation [37], we speculate that these metabolic changes not only reflect differences in nodules but may also stem from systemic changes triggered by strain LL2. Future studies should further dissect the contribution of nodule biomass effects versus intrinsic metabolic regulation to host photosynthetic metabolism by conducting tissue-specific metabolomic analyses of isolated nodules.

Plants possess multiple respiratory metabolic pathways, including glycolysis, the tricarboxylic acid cycle, and the pentose phosphate pathway [38–40]. In this study, concerning respiratory metabolism, the levels of respiration‑related metabolites—such as 3‑Phosphoglyceric acid and glucose‑6‑phosphate—in alfalfa roots and nodules increased significantly after inoculation with the LL2 strain. This may be attributed to the enhancement of plant respiratory metabolism by LL2 to meet the high energy demand of nitrogen fixation. In contrast, inoculation with the QL5 strain led to only minor metabolic changes across alfalfa organs, consistent with its low nitrogen‑fixation capacity and weak symbiotic effect. The accumulation of lactic acid in glycolysis and pyruvic acid metabolism observed under QL5 treatment [41] suggests a shift toward anaerobic respiration. Drew MC [42] noted that even under well‑aerated conditions, certain plant tissues (e.g., nodules) may still undergo anaerobic respiration due to local hypoxia in the microenvironment. This phenomenon may be linked to insufficient energy supply resulting from the low nitrogen‑fixation efficiency of QL5, leading to disrupted energy metabolism in the host plant and a reliance on anaerobic respiration for energy production.

In addition to respiratory metabolism, other metabolic pathways may also be involved in the differential responses induced by strains LL2 and QL5. For example, secondary metabolites such as flavonoids play important roles in plant-rhizobia interactions [43], while hormone signaling pathways, including auxin, cytokinin, and ethylene, coordinately regulate the nodulation process [44]. Future studies should further integrate transcriptomic and metabolomic data to systematically dissect the synergistic regulatory mechanisms among respiratory metabolism, secondary metabolism, and hormone signaling networks in efficient nitrogen-fixing systems.

### Relationship between differential metabolites and photosynthetic‑respiratory metabolism, nitrogen fixation capacity, and plant growth in alfalfa

Through metabolomics analysis, this study identified a large number of differential metabolites and conducted KEGG functional annotation and enrichment analysis. The results indicate that differential metabolites across different organs were primarily annotated and enriched in metabolic pathways—including photosynthetic and respiratory metabolism—as well as in amino acid metabolism and nucleotide metabolism.

The accumulation patterns of these differential metabolites are closely linked to plant physiological functions [45]. For instance, following inoculation with the LL2 strain, the content of photosynthesis-related metabolites—such as ADP—in alfalfa leaves increased significantly, a change that may be associated with enhancing photosynthetic efficiency. Additionally, after LL2 inoculation, the levels of amino acids and their derivatives in various alfalfa organs rose notably, likely due to the strain promoting nitrogen uptake and utilization in the plant.

Amino acids are essential for plant growth and development. Their increased content could contribute to both plant growth and stress resistance [46]. Rhizobium inoculation changes the nitrogen metabolism and carbon allocation of host plants through nitrogen fixation. Furthermore, inoculation with the LL2 strain was associated with a significant increase in the content of nucleotides and their derivatives in various alfalfa organs. This may be linked to the ability of the LL2 strain to enhance photosynthetic metabolism and drive abundant carbohydrate production [47].

### Relationship between nodulation‑nitrogen fixation capacity and the regulation of photosynthetic and respiratory metabolism

The nodulation and nitrogen‑fixation capacity of rhizobia is closely linked to plant metabolic regulation [48]. In this study, the LL2 strain—an efficient symbiotic strain—significantly enhanced nodulation and nitrogen fixation in alfalfa, which was associated with widespread changes in plant metabolism [49]. For instance, after LL2 inoculation, the levels of glucose‑6‑phosphate, D‑mannitol‑1‑phosphate, and Trehalose‑6‑phosphate in alfalfa nodules increased markedly, which may contribute to the strain’s ability to promote nodule formation and nitrogen fixation. Moreover, following LL2 inoculation, the content of respiratory‑related metabolites such as 3‑Phosphoglyceric acid rose significantly in alfalfa nodules, suggesting that part of the energy required for nitrogen fixation is supplied by host respiratory metabolism [41]. In contrast, inoculation with the QL5 strain led to a notable accumulation of lactic acid in alfalfa roots and nodules, which may reflect its lower nitrogen‑fixation efficiency and weaker symbiotic performance. These findings indicate that the nodulation and nitrogen‑fixation capacity of rhizobia is likely achieved by modulating the host plant’s metabolic network, involving multiple pathways such as photosynthetic metabolism, respiratory metabolism, and amino acid metabolism.

Beyond changes in metabolite levels, the structural and developmental characteristics of nodules themselves may contribute to these metabolic differences. Nodule type and bacteroid development status can significantly influence host metabolic processes [50]. Nodules induced by strain LL2 exhibited typical indeterminate morphology with bifurcated shapes, distinct apical meristems, and smooth, pink-colored surfaces, indicating well-developed bacteroids and high leghemoglobin activity. Although nodules induced by strain QL5 were also indeterminate, they were smaller in size and showed reduced meristematic activity, which may lead to a lower proportion of functional bacteroids and limited nitrogenase activity.

This study found that the efficient symbiotic strain LL2 and the inefficient combination QL5 induced significantly different metabolite accumulation patterns in alfalfa, and these responses exhibited clear tissue specificity. Strain LL2 primarily upregulated photosynthesis-related metabolites in leaves, indicating that it promoted photosynthetic carbon fixation and sucrose synthesis; simultaneously, it upregulated respiratory intermediates (e.g., 3-phosphoglyceric acid) in roots and nodules, suggesting enhanced respiratory metabolism to provide sufficient ATP and carbon skeletons for the nitrogen fixation process. This synergistic pattern of enhanced aboveground photosynthetic product synthesis and belowground respiratory energy production may be achieved through systemic signaling, thereby supporting the efficient symbiosis of LL2.

In contrast, metabolic changes observed under QL5 treatment were relatively minor, mainly characterized by the accumulation of lactate in roots or nodules, implying enhanced anaerobic respiration. The causes of lactate accumulation may be multifaceted: in addition to insufficient energy supply due to the lower nitrogen fixation efficiency of QL5, it may also involve local stress responses induced by QL5, or impaired carbon partitioning of photosynthetic products to nodules resulting in insufficient glycolytic substrates [51]. Drew MC [41] noted that even under generally well-aerated conditions, certain plant tissues (such as nodules) may undergo anaerobic respiration due to localized micro-environmental hypoxia. The metabolic disruption induced by QL5 may reflect the host plant’s energy metabolic adaptation or stress response under inefficient symbiotic conditions.

Regarding the connection between metabolic changes and growth performance, the enhanced photosynthetic carbon fixation and respiratory energy metabolism following LL2 inoculation provide more abundant carbon skeletons and ATP for plant growth, which is consistent with its trend toward higher biomass accumulation. In contrast, the metabolic disruption induced by QL5 may lead to reduced energy utilization efficiency, thereby limiting plant growth and potential yield. These findings indicate that rhizobial strains, by reshaping the host plant’s metabolic network, not only affect local tissue metabolism but ultimately translate into differences in crop productivity. These results indicate that the enhanced respiratory metabolism associated with LL2 may provide sufficient ATP for the nitrogen fixation, whereas the lactate accumulation under QL5 treatment instead reflects a disruption of energy metabolism or the onset of stress responses under an ineffective symbiosis.

Based on the metabolic changes observed in this study, we constructed a schematic model (Fig. 11) to systematically illustrate the effects of inoculating with strains LL2 and QL5 on the alfalfa metabolic network. This model visually presents the relationships among different symbiotic effects, metabolic responses, symbiotic efficiency, and crop productivity, providing a theoretical basis for symbiotic breeding in alfalfa. However, the metabolomic data presented here are correlative and do not establish direct causation. As such, our interpretations are proposed as testable hypotheses for future investigation.

## Conclusion

Rhizobium strain LL2 is an efficient matching strain of Gannong No.9 alfalfa, and QL5 is an inefficient matching strain. After inoculation with the two strains, amino acids and their derivatives, organic acids, and benzene and its derivatives represented the largest proportion of differential metabolites in all alfalfa organs. These metabolites were primarily annotated and enriched in pathways related to photosynthesis and respiratory metabolism. In leaves, seven metabolites were commonly up-regulated in photosynthesis and respiration. Notably, the common metabolite ADP showed significantly higher content under LL2 treatment compared to CKL and QL5. In roots, nine metabolites were differentially up-regulated. Specifically, LL2 treatment elevated the levels of 3-Phosphoglyceric acid and Uridine-5’-diphosphate-glucose (photosynthesis-related), as well as (2 S)-2-Isopropylmalate and L-Glutamic acid (respiration- and nitrogen metabolism-related). In nodules, Trehalose-6-phosphate and alpha-D-glucose-6-phosphate, key nodes in glucose and pentose phosphate metabolism, were present at significantly higher levels than in QL5. Certain metabolites function across multiple pathways. ADP and alpha-D-glucose-6-phosphate connect photosynthesis, respiration, and symbiotic metabolism. 3-Phosphoglyceric acid links photosynthesis with symbiotic metabolism, while L-Glutamic acid and (2 S)-2-Isopropylmalate connect respiration with symbiotic metabolism.

Therefore, the high nitrogen-fixing efficiency associated with the LL2 strain may be linked to its role as a “multi-pathway metabolic coordinator,” rather than a regulator of a single symbiotic process. The observation that key node metabolites were co-upregulated in parallel with higher nitrogenase activity suggests a model wherein photosynthetic carbon fixation, pentose phosphate pathway flux, respiratory energy output, and nitrogen metabolism undergo systematic modulation. The resulting co-occurring enhancement in energy supply and accumulation of metabolic precursors for the plant jointly drive higher nitrogenase activity and biomass accumulation. This finding is of great significance for enhancing nitrogen accumulation by regulating the energy strategy of the symbiotic system, improving the nitrogen fixation efficiency of the legume symbiotic system, and increasing the yield and quality of legume crops.

## Supplementary Information

Supplementary Material 1.

Supplementary Material 2: Supplementary Fig. 1 Pathway classification and enrichment analysis of differential metabolites in leaves. (a) Classification map of differential metabolic pathways in QL5L vs CKL. (b) Enrichment map of differential metabolic pathways in QL5L vs CKL. (c) Classification map of differential metabolic pathways in LL2L vs QL5L. (d) Enrichment map of differential metabolic pathways in LL2L vs QL5L. Supplementary Fig. 2 Pathway classification and enrichment analysis of differential metabolites in roots. (a) Classification map of differential metabolic pathways in QL5R vs CKR. (b) Enrichment map of differential metabolic pathways in QL5R vs CKR. (c) Classification map of differential metabolic pathways in LL2R vs QL5R. (d) Enrichment map of differential metabolic pathways in LL2R vs QL5R.

## Data Availability

The data presented in this study are available on request from the corresponding authors.
