# Supplementary material for: Efficient rhizobium strains enhance nitrogen fixation and growth in alfalfa by improving photosynthetic carbon metabolism and respiratory nitrogen assimilation
Source: BMC Plant Biol. 2026 May 26;26:1069. doi: 10.1186/s12870-026-08954-4 (PMC13285294; doi:10.1186/s12870-026-08954-4)
Supplement: Supplementary file 2 — Supplementary Material 2: Supplementary Fig. 1 Pathway classification and enrichment analysis of differential metabolites in leaves. (a) Classification map of differential metabolic pathways in QL5L vs CKL. (b) Enrichment map of differential metabolic pathways in QL5L vs CKL. (c) Classification map of differential metabolic pathways in LL2L vs QL5L. (d) Enrichment map of differential metabolic pathways in LL2L vs QL5L. Supplementary Fig. 2 Pathway classification and enrichment analysis of differential metabolites in roots. (a) Classification map of differential metabolic pathways in QL5R vs CKR. (b) Enrichment map of differential metabolic pathways in QL5R vs CKR. (c) Classification map of differential metabolic pathways in LL2R vs QL5R. (d) Enrichment map of differential metabolic pathways in LL2R vs QL5R. [file 12870_2026_8954_MOESM2_ESM.docx]

**Supplementary Information**

**
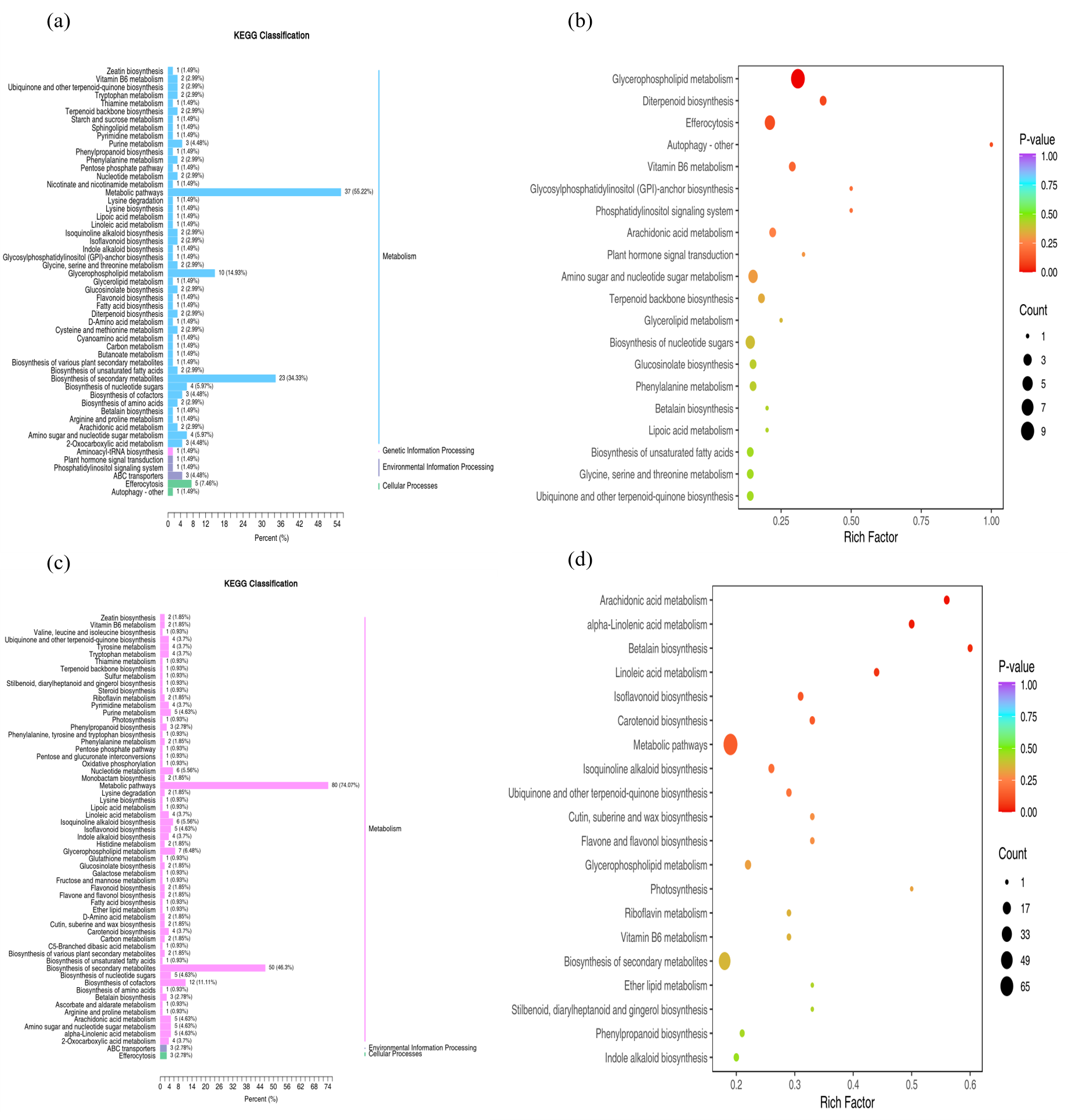
**

**Supplementary Fig. 1** Pathway classification and enrichment analysis of differential metabolites in leaves. (a) Classification map of differential metabolic pathways in QL5L vs CKL. (b) Enrichment map of differential metabolic pathways in QL5L vs CKL. (c) Classification map of differential metabolic pathways in LL2L vs QL5L. (d) Enrichment map of differential metabolic pathways in LL2L vs QL5L.

**
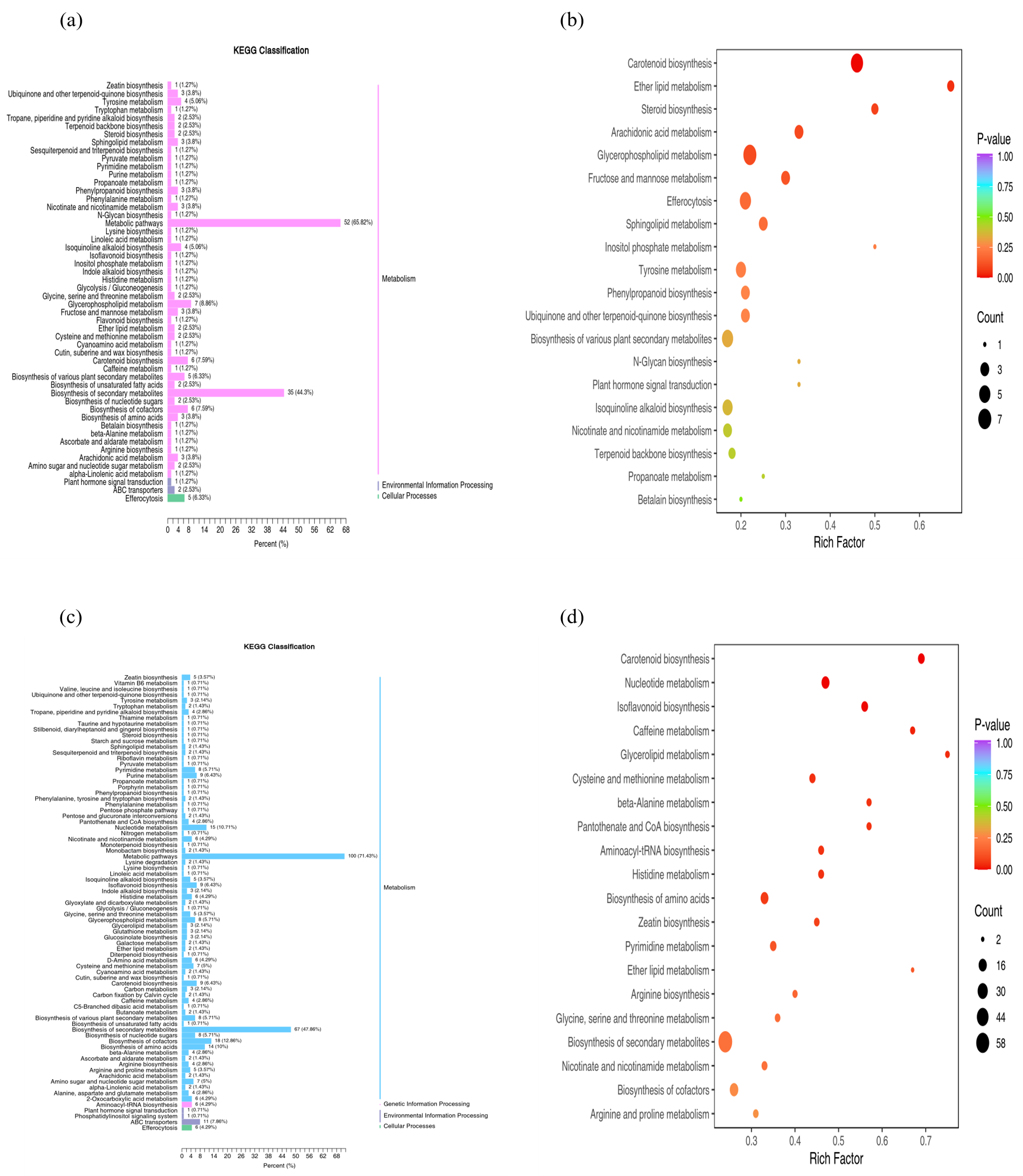
**

**Supplementary Fig. 2** Pathway classification and enrichment analysis of differential metabolites in roots. (a) Classification map of differential metabolic pathways in QL5R vs CKR. (b) Enrichment map of differential metabolic pathways in QL5R vs CKR. (c) Classification map of differential metabolic pathways in LL2R vs QL5R. (d) Enrichment map of differential metabolic pathways in LL2R vs QL5R.
